# Phosphoproteomics of primary AML patient samples reveals rationale for AKT combination therapy and p53 context to overcome selinexor resistance

**DOI:** 10.1016/j.celrep.2022.111177

**Published:** 2022-08-09

**Authors:** Kristina B. Emdal, Nicolàs Palacio-Escat, Caroline Wigerup, Akihiro Eguchi, Helén Nilsson, Dorte B. Bekker-Jensen, Lars Rönnstrand, Julhash U. Kazi, Alexandre Puissant, Raphaël Itzykson, Julio Saez-Rodriguez, Kristina Masson, Peter Blume-Jensen, Jesper V. Olsen

**Affiliations:** 1 Proteomics Program, Novo Nordisk Foundation Center for Protein Research, Faculty of Health and Medical Sciences, University of Copenhagen, Copenhagen, Denmark; 2 Heidelberg University, Faculty of Medicine and Heidelberg University Hospital, Institute for Computational Biomedicine, BioQuant-Zentrum, Heidelberg, Germany; 3 Heidelberg University, Faculty of Biosciences, Heidelberg, Germany; 4 RWTH Aachen University, Faculty of Medicine, Joint Research Centre for Computational Biomedicine, Aachen, Germany; 5 Acrivon Therapeutics Inc., Watertown, MA, USA; 6 Division of Translational Cancer Research, Department of Laboratory Medicine, Lund University, Lund, Sweden; 7 INSERM UMR 944, IRSL, St Louis Hospital, Paris, France

**Keywords:** phosphoproteomics, mass spectrometry, functional scoring, selinexor, acute myeloid leukemia, drug resistance, combination therapy, MK-2206, subcellular proteomics, nutlin-3a

## Abstract

Acute myeloid leukemia (AML) is a heterogeneous disease with variable patient responses to therapy. Selinexor, an inhibitor of nuclear export, has shown promising clinical activity for AML. To identify the molecular context for monotherapy sensitivity as well as rational drug combinations, we profile selinexor signaling responses using phosphoproteomics in primary AML patient samples and cell lines. Functional phosphosite scoring reveals that p53 function is required for selinexor sensitivity consistent with enhanced efficacy of selinexor in combination with the MDM2 inhibitor nutlin-3a. Moreover, combining selinexor with the AKT inhibitor MK-2206 overcomes dysregulated AKT-FOXO3 signaling in resistant cells, resulting in synergistic anti-proliferative effects. Using high-throughput spatial proteomics to profile subcellular compartments, we measure global proteome and phospho-proteome dynamics, providing direct evidence of nuclear translocation of FOXO3 upon combination treatment. Our data demonstrate the potential of phosphoproteomics and functional phosphorylation site scoring to successfully pinpoint key targetable signaling hubs for rational drug combinations.

## Highlights

• Phosphoproteomics with functional scoring uncovers context for selinexor sensitivity
• Functional p53 correlates with selinexor sensitivity, which is enhanced by nutlin-3a
• Dysregulated AKT-FOXO3 drives selinexor resistance, which is overcome with MK-2206
• Spatial proteomics reveals selinexor-induced nucleocytoplasmic protein shuttling

## Graphical abstract

**Figure:**
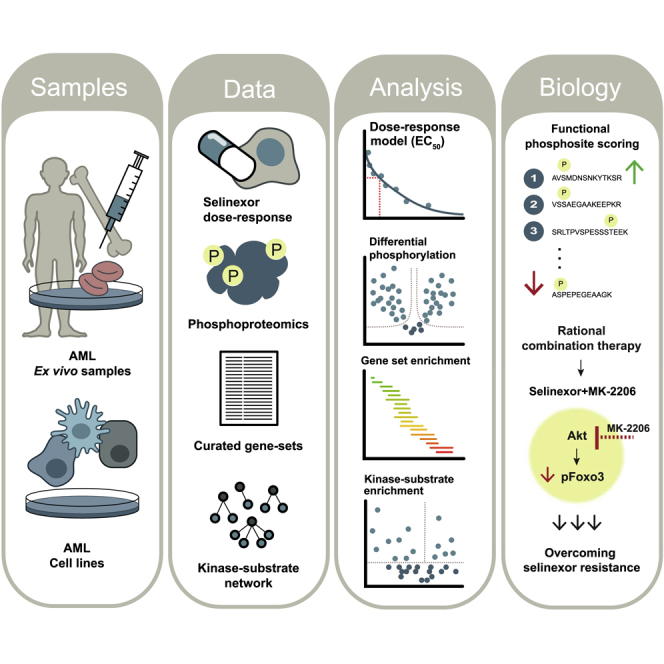

## Teaser

Emdal et al. combine phosphoproteomics of samples from patients with AML and functional phosphosite scoring to uncover clinically actionable molecular context for selinexor efficacy. Sensitivity to selinexor correlates with functional p53 and is enhanced with nutlin-3a, while resistance is associated with dysregulated AKT-FOXO3 signaling and overcome by combining with MK-2206.

## Introduction

Acute myeloid leukemia (AML) is the most common acute leukemia in adults and is characterized by disease heterogeneity, both phenotypically and genetically (Prada-Arismendy et al., 2017). The repertoire has expanded into numerous different molecularly defined subtypes (Papaemmanuil et al., 2016), underscoring the complexity of the genetic landscape. The standard-of-care therapy for AML includes cytotoxic chemotherapy interfering with DNA and RNA synthesis; e.g. cytarabine and anthracyclines (Rai et al., 1981) for patients fit enough to receive these treatments. Sadly, these treatment regimens have not advanced significantly for decades, and targeted therapies for AML are limited (Vetrie et al., 2020). Only within the past few years have successful clinical trials and regulatory approvals of targeted therapies for AML advanced the field toward precision oncology (Daver et al., 2019; Wei and Tiong, 2017). Nevertheless, despite initial response to conventional therapies, the long-term prognosis for AML patients is sub-optimal, with a relapse rate of ∼50% and a 5-year overall survival rate of ∼30% (Medeiros et al., 2019). A universal challenge in cancer therapy relates to understanding of the underlying molecular mechanisms of *de novo* or acquired therapy resistance with the premise of new avenues for targeted therapeutic approaches, including for AML.

Selective inhibitors of nuclear export (SINE) hold promise as a potential therapeutic strategy for overcoming resistance to conventional chemotherapy in AML (Pardee et al., 2020; Sweet et al., 2020; Talati and Sweet, 2018). The first-in-class SINE compound, selinexor, reversibly binds to and inhibits the nuclear export protein exportin-1 (XPO1), leading to accumulation of cargo proteins in the cell nucleus (Fung and Chook, 2014; Thakar et al., 2013). These include tumor suppressor proteins; e.g., p53, cell cycle regulators, and targets of chemotherapeutic agents such as topoisomerases (Alt et al., 2002; Subhash et al., 2018; Turner et al., 2013). XPO1 is overexpressed in AML cells, and increased levels of XPO1 are inversely correlated with overall survival in AML patients (Kojima et al., 2013). Moreover, in preclinical AML models, selinexor has demonstrated encouraging anti-leukemic effects (Etchin et al., 2013; Ranganathan et al., 2012), spurring a surge in clinical trial testing of selinexor as single agent or in combination with other therapies in numerous solid and liquid tumor trials, including at least 15 trials in AML (clinicaltrials.gov). Studies so far indicate limited single-agent activity in AML, while combination therapies with primarily non-targeted agents show some clinical promise (Bhatnagar et al., 2020; Wang et al., 2018). However, targeted inhibitors of cellular signal transduction pathways provide a hitherto minimally explored potential for combination with selinexor, which is clinically attractive given the potential for an improved therapeutic index compared with chemotherapy combinations.

Mass spectrometry (MS)-based quantitative phosphoproteomics is a powerful method for global analysis of signaling networks, and the technology has proved valuable in identifying targetable and drug-regulated signaling nodes in various disease models (Andersen et al., 2010; Emdal et al., 2018; Francavilla et al., 2017; Lescarbeau et al., 2016; Rikova et al., 2007). To uncover clinically actionable, rational drug combinations with selinexor in AML, we performed MS-based quantitative phosphoproteomics analyses of the response to selinexor using *ex vivo* primary AML patient samples and cell lines. We characterized the phosphorylation events associated with selinexor sensitivity and resistance using functional scoring of phosphosites (Ochoa et al., 2020), an approach termed phosphoproteomics analysis with functional scoring (PAFS), to identify key signaling nodes of biological importance. Based on PAFS, we found that functional p53 context provides single-agent selinexor sensitivity, which is further enhanced with nutlin-3a, while activated AKT-forkhead box O3 (FOXO3) survival signaling is a key selinexor resistance mechanism that can be overcome by combining selinexor with an AKT inhibitor. Using spatial proteomics, we further demonstrated this mechanism to involve cytoplasmic to nuclear translocation of FOXO3 upon the combination therapy, consistent with the release of FOXO3 from cytoplasmic sequestration by 14-3-3 proteins upon AKT inhibition.

## Results

### Selinexor response in *ex vivo*-cultured primary human AML cells

**Figure 1:**
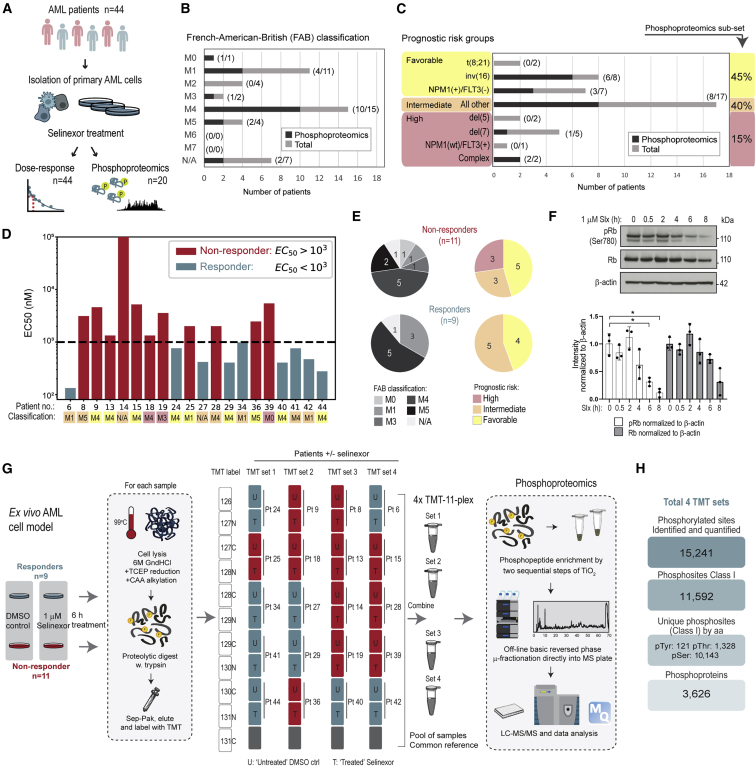
Phosphoproteomics analysis of the selinexor response in AML *ex vivo* samples (A) Schematic representation of the workflow for characterization of the selinexor response in *ex vivo*-treated AML patient samples (n = 44). (B and C) Patient distribution in cohort according to the French-American-British (FAB) subtype classification of AML morphology (B) and the European LeukemiaNet 2017 risk stratification model by genetic abnormality (C). (D) Selinexor EC50 values for the 20 *ex vivo* AML patient samples in the phosphoproteome analysis with grouping into selinexor responders (EC50 < 1,000 nM; n = 9) and non-responders (EC50 > 1,000 nM; n = 11). See also Figure S1. (E) Distribution of selinexor non-responders and responders according to FAB classification (left) and risk stratification by genetics (right). (F) Western blot analysis (upper) and quantification (lower) of phospho-Rb (S780) and Rb levels in MV-4-11 cells after treatment with selinexor for 0.5–8 h. Data in bar graph represent mean ± SD (n = 3 independent experiments). ^∗^p < 0.05 by two-sample Student’s t test with Bonferroni correction. (G) Experimental workflow of the MS-based quantitative phosphoproteome analysis of the selinexor response in AML cells isolated from 20 patients presented in (D). (H) Summary of phosphoproteome data with the number of identified phosphorylation sites and phosphoproteins. See also Figures S1–S3; Tables S1 and S2.

To characterize the response to selinexor in a patient-derived *ex vivo* model, we isolated blasts from either bone marrow or peripheral blood from 44 treatment-naive AML patients (Figure 1A; Table S1). The patient samples showed good coverage of different AML subtypes (M0–M5) according to the French-American-British (FAB) classification system (Bennett et al., 1976), with most samples being either M1 or M4 as expected from a treatment-naive population (Figure 1B). Moreover, all AML risk groups based on the European LeukemiaNet (ELN) 2017 risk stratification model (Döhner et al., 2017) were represented (Figure 1C). Hence, we deemed the sample collection to cover an expected spectrum of AML heterogeneity and be reasonably representative of the general population.

*Ex vivo*-cultured patient AML cells were treated with selinexor (Figure 1A) and, based on cell viability measurements 48 h post treatment, dose-response curves were generated and fitted dose-response models derived from a Hill function (Figure S1; Table S1). The values for half-maximal effective concentration (EC50) were determined for each patient sample and a binary cutoff of 1 μM was chosen to distinguish selinexor responders (EC50 < 1 μM) from non-responders (EC50 > 1 μM) (Table S1). Among the 44-patient cohort, we identified 18 (40.9%) responders and 26 (59.1%) non-responders to selinexor treatment based on this cutoff.

### Phosphoproteomics analysis of the selinexor response in *ex vivo* AML patient samples

To study the signaling events associated with selinexor response in AML, we performed quantitative MS-based phosphoproteomics profiling of 20 of the 44 patient samples, including nine responders and 11 non-responders (represented in Figure 1D), for which sufficient protein amounts of 50–100 μg could be isolated. For this subset of samples, AML heterogeneity was evident in terms of subtype classification except for the more differentiated M2 subtype (Figure 1B) and the prognostic risk groups covered 45% favorable, 40% intermediate, and 15% high-risk patients (Figure 1C). The selinexor non-responders showed more diversity in terms of FAB classification and risk grouping compared with the responders, which were mainly the more common M1 and M4 subtypes with intermediate/favorable risk groups (Figure 1E). Prior to phosphoproteomics analysis, an optimal time point for selinexor stimulation was determined by treating sensitive MV-4-11 human AML cells with 1 μM selinexor and monitor the relative phosphorylation of S780-Rb for up to 0.5–8 h. The tumor suppressor Rb is a known, functionally important cargo protein of XPO1, and selinexor treatment has been shown to reduce the cyclin-dependent kinase (CDK) 4/6/cyclin D1-dependent inactivating phosphorylation of Rb (Baek et al., 2018). We deemed the 6-h time point as optimal based on reduced phosphorylation with limited effect on total Rb levels (Figure 1F) (Wang and Liu, 2019). Subsequently, *ex vivo*-cultured AML cells from each patient were treated with selinexor or DMSO and the samples processed according to a workflow based on tandem mass tag (TMT) labeling for precise quantitation as previously described (Emdal et al., 2018), and analyzed as outlined in Figure 1G. From the phosphoproteome analysis, we identified and quantified 15,241 phosphorylated sites, 11,592 of which were localized with high confidence (class I; Olsen et al., 2006) and covered 3,626 phosphoproteins (Figures 1H, S2A, and S2B; Table S2).

To characterize the global effects of selinexor on the phosphoproteome, TMT reporter ion intensities from each TMT11-plex dataset were normalized using the variance stabilization normalization (VSN) method (Huber et al., 2002) and batch effect corrected (Figures S2C and S2D). We assessed the level of regulation by differential phosphorylation site analysis and, based on the resulting volcano plots (Figure S2E), we identified 1,023 downregulated and 994 upregulated by selinexor as well as the regulation of phosphosites in the responders (downregulation, 745; upregulation, 720) and non-responders (downregulation, 221; upregulation, 335). The lower number of regulated phosphorylation sites in the non-responders suggests that selinexor induced weaker effects on the phosphoproteome in patients resistant to selinexor.

Overrepresentation analysis of sequence motifs among the regulated phosphorylation sites in responders and non-responders revealed no major differences between the two groups (Figure S2F). However, a strong preference for proline residues in the +1 position to the phosphorylated residue, which is characteristic for proline-directed kinases (e.g., mitogen-activated protein kinases [MAPKs] and CDKs), and a preference for the R-X-X-pS/T motif, characteristic for PKA/B/C kinases, was observed irrespective of directionality up or down of phospho-regulation. A high frequency of glutamine (Q) in the +1 position was also observed, specifically for upregulated phosphorylation sites in responder and non-responder groups. This is the preferred consensus motif for DNA damage response (DDR) pathway kinases, including ataxia telangiectasia mutated (ATM) and ataxia telangiectasia and Rad3 related (ATR), suggesting increased signaling due to activation of DNA-damage stress responses. Moreover, kinase-substrate enrichment analysis (KSEA) (Figures S3A–S3D) showed that the CSNK(1/2)A1 substrate motif was enriched in treated non-responders (Figure S3C) and the cell cycle regulator WEE1 appeared activated in non-responders compared with responders prior to treatment (Figure S3D).

Based on the statistical significance (p values) and directionality (up or down) of the differentially selinexor-regulated phosphorylation sites, we performed a gene set enrichment analysis (GSEA) of the phosphoproteins. The top enriched gene sets show a clear consensual upregulation of transcription and RNA processing and splicing in both responder and non-responder samples (Figure S3E). In the case of top enriched downregulated gene sets, there were both shared sets and some unique sets for responders and non-responders. Among the shared downregulated gene sets are deubiquitinases of the Ub C-terminal hydrolase (UCH) proteinase family; insulin, and vascular endothelial growth factor (VEGF) pathway, a possible indication of early triggered apoptosis (onset of protein degradation and suppression of growth and proliferating signals). JAK-STAT, innate immune system, and B cell receptor signaling are gene sets uniquely downregulated in non-responders, which potentially could be related to resistance mechanisms.

### Genetic annotation of patient samples shows poor correlation with selinexor response

Next-generation genomic sequencing of AML patient samples has revealed major genetic diversity with several driver mutations and distinct molecular subgroups. A comprehensive characterization of the genomic landscape in AML was previously described (Papaemmanuil et al., 2016). To explore whether response to selinexor correlated with specific genomic subtypes, we performed a basic computational analysis of the reported genomic aberrations, including FLT3 status and WT1 expression. Comparing the overlap between samples of different genetic annotations and their response to selinexor using the Szymkiewicz-Simpson index or overlapping coefficient, we found limited correlation between FLT3 internal tandem duplication (ITD) as well as mutations in NPM1 (types A and G) with lack of response to selinexor (Figure S3F). However, this correlation was not exclusive and therefore non-conclusive. In conclusion, we did not observe a clear and distinct correlation between known genomic aberrations in the established genomic AML subtypes and sensitivity to selinexor.

### Functional scoring of phosphorylation sites identifies key signaling rewiring

**Figure 2:**
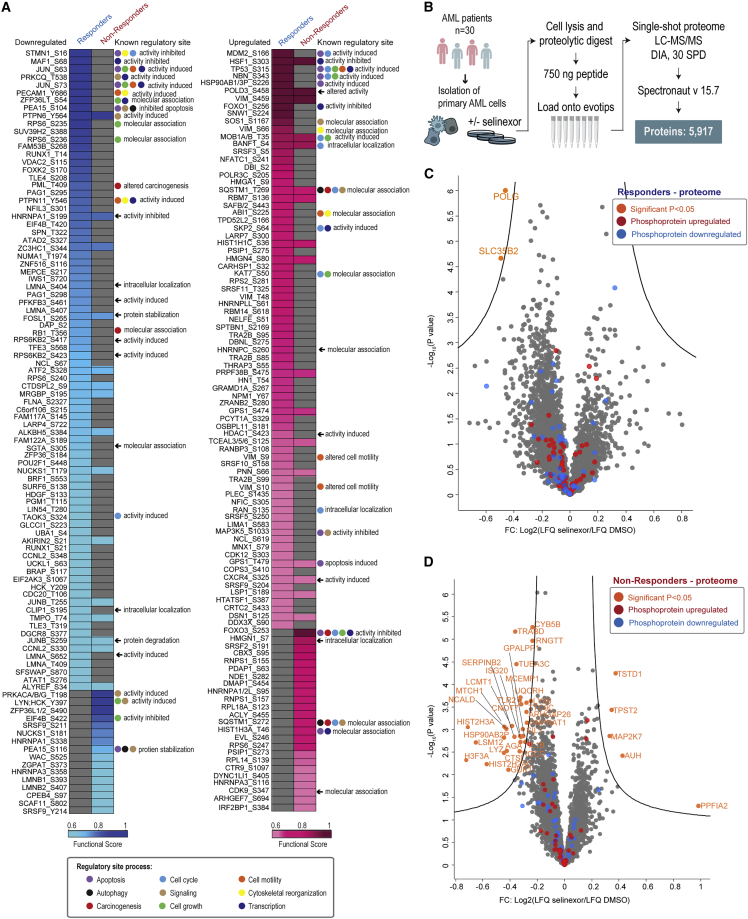
Functional scoring of phosphorylation sites identifies key signaling rewiring in response to selinexor (A) Overview of the functional sites assigned to phosphorylation sites identified to be down- (left) or upregulated (right) among responders and non-responders in response to selinexor treatment. Phosphorylation sites with a functional score >0.6 are represented. Known regulatory site functions and their involvement in a biological process as indicated by colored circles are functionally annotated based on PhosphoSitePlus (Hornbeck et al., 2015) and retrieved from Perseus software (Tyanova et al., 2016). (B) Workflow for single-shot MS-based proteome analysis of 30 *ex vivo* AML patient samples. (C and D) Volcano plots showing differentially regulated proteins from the single-shot proteome MS analysis. Fold change represents selinexor treatment versus DMSO control of *ex vivo*-grown AML cells for responders (n = 8) (C) and non-responders (n = 22) (D). Significance was deemed based on a two-sided t test (FDR < 0.05, s0 = 0.1) using Perseus software and highlighted in orange coloring. Red and blue coloring indicate the proteins with regulated phosphosites shown in (A). See also Figures S4 and S5; Tables S2 and S3.

To pinpoint the most biologically relevant phosphorylation sites within the regulated phosphoproteome (Figure S2E), and, thus, key players in the pharmacodynamic and adaptive response to selinexor, we applied functional scoring with PAFS (Ochoa et al., 2020) to each significantly drug-regulated phosphorylation site among the responders and non-responders. In the work by Ochoa et al., the functional scoring is based on a machine learning (ML) approach applied to 112 large-scale public phosphoproteome datasets from the PRIDE database. Here, integration of prior knowledge from experimental data, prediction tools, and MS signal properties was used to generate a functional score (between 0 and 1), which allowed the ranking of phosphosites according to biological relevance (Ochoa et al., 2020). Figure 2A lists the top-scoring (score > 0.6) phosphorylation sites significantly regulated by selinexor in responders (down, 83; up, 76) and non-responders (down, 31; up, 41) with minimal overlap between groups (Figure S4A). The top five downregulated sites scored in responders were the transcriptional repressor MAF1 S68, stathmin 1 (STMN1) S16, the transcription factor Jun S63 and S73, and protein kinase C theta (PRKCQ) T538. Conversely, the top five downregulated sites scored in non-responders were cyclic AMP-dependent protein kinase catalytic subunit α/β/γ (PRKACA, PRKACB, PRKACG) T198, SRC family kinase LYN/HCK Y397/Y411, ZFP36L1/L2 S334/S490, the translation initiation factor EIF4B S422, and splicing factor SRSF9 S211 (Figure 2A). The highest-scored upregulated sites in responders include the transcription factor heat shock factor (HSF1) S303, the tumor suppressor p53 S315, and its E3 ubiquitin ligase MDM2 S166. Phosphorylation of the p53 site provides a docking motif for PIN1, which regulates p53 stability and transcriptional activity in response to DNA damage (Liu et al., 2019; Wulf et al., 2002). These findings suggest that p53 enhancement could potentiate the activity of selinexor.

In contrast, the highest scoring site in non-responders was FOXO3 S253 (Figure 2A) and phosphorylation of this site by AKT has been shown to retain FOXO3 sequestrated in the cytoplasm through binding with 14-3-3 and thus preventing its translocation to the nucleus where it exerts pro-apoptotic transcriptional effects (Brunet et al., 1999). Hence, activation of AKT-FOXO3 survival signaling may serve as a biologically important consequence of selinexor treatment in non-responders. In addition to the FOXO signaling pathway, several components of the mammalian target of rapamycin (mTOR) signaling pathway (e.g., RPS6K2, RPS6, EIF4B, CLIP1) received high functional scores indicating regulation by AKT signaling (Figure 2A). A heatmap display of all regulated phosphorylation sites from proteins assigned to either mTOR or FOXO signaling pathway revealed a high degree of inter- and intra-sample heterogeneity, underscoring the great complexity of drug-regulated pathways (Figure S4B). However, based on our functional phosphosite scoring analysis, we could pinpoint AKT as a kinase with key activity in selinexor-resistant AML cells, immediately generating the testable hypothesis that AKT inhibition with selinexor would, at least partially, overcome this resistance.

To determine whether the observed phosphoproteome effects were associated with proteome changes or true regulation at the phosphorylation-site level, we measured a single-shot proteome (± selinexor treatment; 1 μM, 6 h) for 30 *ex vivo* samples with known selinexor response (Figures S4C and S5; Table S3). We identified and quantified 5,917 proteins (Figure 2B; Table S3) and found that none of the phosphoproteins in responders and non-responders highlighted in Figure 2A showed significant changes at the proteome level (Figures 2C and 2D). Hence, the observed phosphoproteome changes highlighted in Figure 2A were truly due to stoichiometric regulation at the phospho-site level. Limited differences were observed between responders and non-responders at the pre-treatment and post-treatment proteome level (Figures S4D and S4E), while proteome abundance of proteins related to mTOR and FOXO signaling confirmed a high degree of inter- and intra-sample heterogeneity at the proteome level, too (Figure S4F).

### Phosphoproteomics analysis of selinexor response in sensitive and resistant AML cell lines

**Figure 3:**
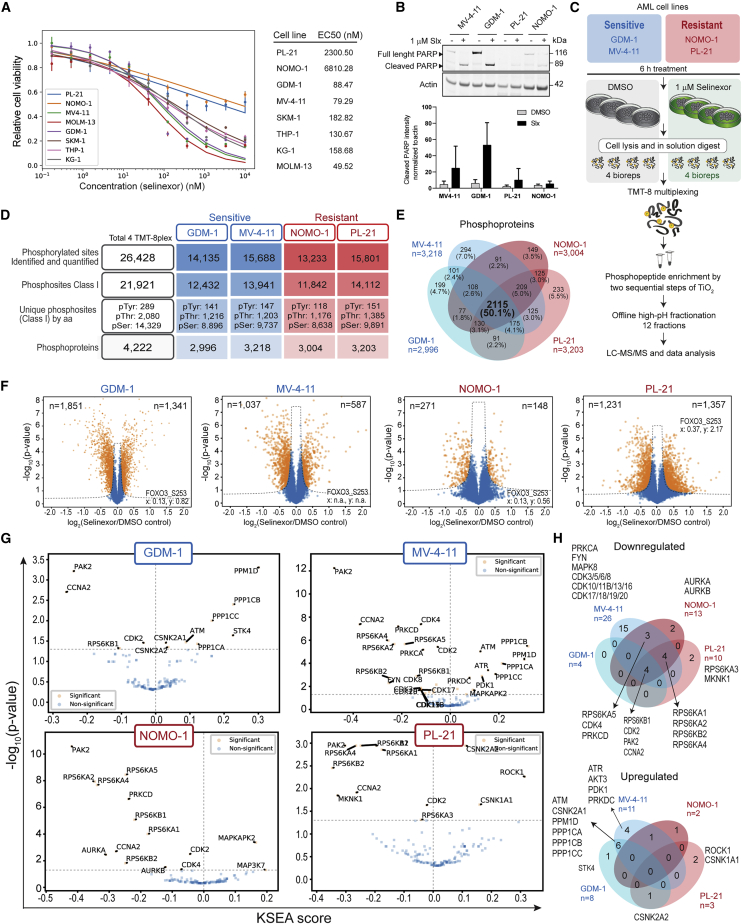
Phosphoproteomics analysis of the selinexor response in sensitive and resistant AML cell lines (A) Dose-response cell viability curves for a panel of AML cell lines treated with selinexor or DMSO as control and estimated relative and absolute EC50 values for each of the cell lines (n = 2–3 independent experiments; n = 4 technical replicates each dose). (B) Lysates from AML cells treated with selinexor or DMSO for 24 h, immunoblotted for cleaved PARP and actin (for reference) (upper) and quantified (lower). Data in bar graph represent mean ± SD (n = 3 independent experiments). (C) Overview of the experimental workflow for quantitative MS-based phosphoproteome analysis of four selected AML cell lines. Each cell line was treated with selinexor or DMSO (n = 4 independent experiments). (D) Summary of phosphoproteome data including the number of identified phosphorylation sites and phosphoproteins derived from the analysis. (E) Overlap between phosphoproteins identified in the AML cell lines; selinexor-sensitive (blue coloring) and -resistant cells (red coloring). (F) Volcano plots showing differentially regulated phosphorylation sites. Fold change represents selinexor treatment versus DMSO control for each AML cell line. Significance was deemed by two-sided t test (n = 4 independent experiments, FDR < 0.05, s0 = 0.1) using Perseus software. (G) Kinase-substrate enrichment analysis (KSEA). Dots in orange with labels are the significantly enriched kinases corresponding to the selinexor-treated versus untreated (DMSO control). Significance was considered for p values <0.05. (H) Overlap in enriched kinases based on KSEA in (G). See also Figure S6; Table S4.

Prior to testing our *ex vivo*-derived hypothesis that dual AKT and XPO1 inhibition in AML cell lines would abrogate selinexor resistance, we initially wanted to confirm the global phosphoproteome findings and subsequently screened a panel of nine AML cell lines for selinexor sensitivity and subjected them to the PAFS analytical approach as well. GDM-1 and MV-4-11 were selected as selinexor sensitive, while NOMO-1 and PL-21 were selected as resistant, based on dose-response curves and calculated EC50 values (Figure 3A). A higher expression of cleaved poly (ADP-ribose) polymerase (PARP) after selinexor treatment in GDM-1 and MV-4-11, compared with NOMO-1 and PL-21, further substantiated that the increased selinexor sensitivity in GDM-1 and MV-4-11 cell lines was consistent with drug-induced apoptosis (Figure 3B). We then analyzed the phosphoproteome after 6 h of selinexor treatment of these four cell lines as outlined in Figure 3C. We identified and quantified 26,428 phosphorylated sites, 21,921 of which were confidently localized to serine (85.8% of the total or 14,329 unique sites), threonine (12.5% or 2,080 unique sites), or tyrosine (1.7% or 289 unique sites) residues in the peptide sequence (class I) within 4,222 proteins with a good protein overlap of 50% between cell lines (Figures 3D and 3E; Table S4). TMT intensities from each of the four TMT sets were normalized and batch effect corrected as described above (Figure S6B). The level of selinexor-regulated sites in each cell line was determined by differential analysis as shown in the volcano plots (Figure 3F). In GDM-1, 1,851 sites were downregulated, and 1,341 sites were upregulated. In MV-4-11, 1,037 sites were downregulated, and 587 were upregulated. In PL-21, 1,231 sites were downregulated and 1,357 were upregulated. In NOMO-1, 271 sites were downregulated and 148 were upregulated.

Sequence motif overrepresentation for each cell line showed a strong preference for proline residues in the +1 position to the phosphorylated residue and a preference for the R-X-X-pS/T motif among the regulated phosphorylation sites (Figure S6C), consistent with the findings observed in the AML *ex vivo* model (Figure S2F). The preference for Q in the +1 position of the phosphosite was only observed in the MV-4-11 cell line, pointing toward potential differences in the DNA-damage response pathway activity between *in vitro* and *ex vivo*-cultured cells, possibly ascribable to the differences in handling prior to treatment, cell culture media differences, or adaptive changes in cell lines grown in long-term culture (Cucchi et al., 2020). The resistant PL-21 cells showed a preference for glutamic acid (E) in the +3 position among upregulated phosphorylation sites as also observed for non-responders in the AML *ex vivo* model. The KSEA revealed a shared core of RPS6KA/B kinase substrates downregulated by selinexor (Figures 3G and 3H) in all groups, which supports the PAFS approach identifying key functional phosphorylation sites of the mTOR signaling pathway; e.g., RPS6 and RPS6K in the *ex vivo* model (Figure 2A).

The GSEA of the selinexor-treated cell lines (Figures S6D–S6E) showed downregulation of cell cycle-related processes, specifically G1 phase-related in agreement with the *ex vivo* model. While processes related to RNA processing were commonly upregulated in the patient-derived samples, some of these gene sets were found to be downregulated in MV-4-11 cells. In the resistant PL-21 cells, several upregulated pathways were related to autophagy, which could potentially be related to selinexor resistance (Zhang et al., 2016). Furthermore, shared upregulated gene sets were related to immune response and viral infection processes.

### Functional scoring of selinexor-regulated phosphosites in AML cell lines recapitulates the diversity in selinexor response

**Figure 4:**
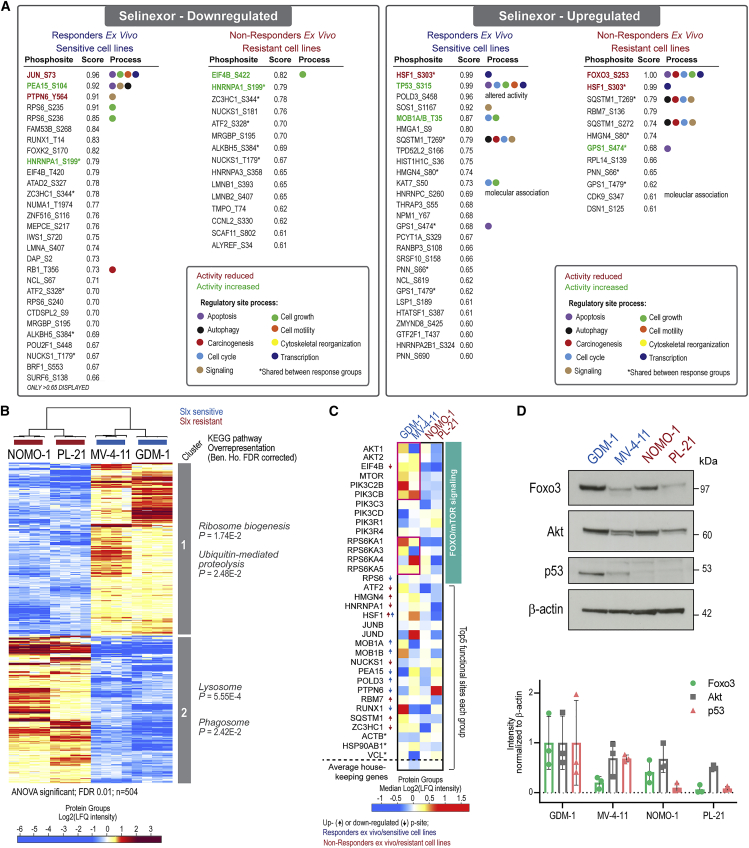
The selinexor responses in the AML *ex vivo* model and cell lines show common and unique signaling responses (A) List of phosphorylation sites with a functional score >0.6 commonly regulated between *ex vivo* analysis and AML cell lines. The list highlights known regulatory site functions based on information obtained from Perseus software. Asterisk (^∗^) indicates shared phosphorylation sites between response groups. (B) Hierarchical clustering of significantly regulated proteins (n = 504; FDR = 0.01, s0 = 0.1) between selinexor-sensitive (blue color) and -resistant (red color) cells. KEGG pathway overrepresentation with Benjamini-Hochberg FDR-corrected p values for cluster 1 and 2. (C) Heatmap of median log2-transformed label-free quantification (LFQ) intensities for a selection of regulated proteins belonging to FOXO3/mTOR signaling from (B) and include the top five most regulated proteins in (A). Arrows indicate the phosphorylation site (p-site) regulation directionality in (A) and blue/red colored text refers to the selinexor response group. Asterisk (^∗^) indicates housekeeping genes. (D) Western blot analysis (upper) and quantification (lower) of FOXO3, AKT, and p53 in AML cells. Data in bar graph represent mean ± SD (n = 3). See also Figure S7; Tables S2, S3, and S4.

Next, we compared the selinexor-regulated phosphoproteome from the primary *ex vivo* models with the established cell line models. Of note, approximately half of the drug-regulated phosphosites (45%–53%) regulated in the *ex vivo* model were also regulated in the cell line models (Figure S7A). Overall, we were able to detect a significantly larger number of regulated phosphosites in cell lines than in the *ex vivo* model. We and others have observed similar discrepancies in studies comparing cell lines with patient samples, which likely reflects the availability of more total protein from cell lines and the potential impact of cell-line-specific adaptation to growth in 2D. In an attempt to uncover the most important pathways that were regulated in a similar manner in both models, we performed PAFS on the regulated phosphoproteome of cell lines and ranked the highest scoring (>0.6) functional phosphorylation sites shared with the *ex vivo* models (Figure 4A). A few phosphosites were downregulated by drug treatment irrespective of response group; e.g., S199 of HNRNPA1, a protein involved in processing and transport of mRNA reported to functionally interact with XPO1 (la Cour et al., 2004). Accordingly, S199 phosphorylation is a potentially attractive pharmacodynamic drug target engagement biomarker for selinexor. Conversely, the HSF1 S303 site was upregulated by drug treatment in both response groups. Of note, the majority of listed functional sites were regulated oppositely by selinexor in sensitive and resistant cells. In *ex vivo* responders/sensitive cell lines, the sites of JUN S73, PEA15 S104, PTPN6 Y564, and RPS6 S235/236 with roles in apoptosis, signaling, and cell growth were downregulated, which is consistent with a drug response affecting cell viability. Among the upregulated sites in responders/sensitive cells, we observed a high functional score for p53 S315. In non-responders/resistant cells, we observed downregulation of EIF4B S422 phosphorylation, a known phosphorylation site convergently targeted by several signaling cascades involved in tumorigenesis, including the core PI3K/AKT/MTOR signaling axis (Chen et al., 2016; Raught et al., 2004; Shahbazian et al., 2006). Interestingly, the phosphorylation site upregulated with the highest scoring in non-responders/resistant cells is FOXO3 S253, a key inhibitory site that may suggest inhibition of apoptosis as a key resistance mechanism in cell lines as well as patient samples. These data suggest the potential for dual AKT and XPO1 inhibition to overcome selinexor resistance.

Single-shot proteome analysis of the sensitive and resistant cell lines was performed as previously described (Batth et al., 2019). We identified and quantified 4,765 proteins (Figure S7B) and showed, among the 504 differentially expressed proteins (ANOVA significant; FDR = 0.01), overrepresentation of proteins related to the Kyoto Encyclopedia of Genes and Genomes (KEGG) terms ribosome biogenesis and ubiquitin-mediated proteolysis among sensitive cells and lysosome and phagosome overrepresentation for resistant cells (Figure 4B). Abundance heterogeneity was evident for the top five most regulated proteins in Figure 4A, and a trend of higher levels of proteins related to mTOR and FOXO3 signaling was observed for sensitive cells (Figure 4C). We further examined the relative levels of AKT, FOXO3, and p53 across the four cell lines by western blotting (Figure 4D). In contrast to the functional correlation between phosphorylation status of these proteins and selinexor sensitivity, there was no correlation between pan protein levels of AKT and FOXO3 and selinexor sensitivity, while increased level of pan p53 correlated with selinexor sensitivity independently of *TP53* mutational status (wild-type *TP53*, GMD-1, MV-4-11, PL-21; and mutated *TP53*, NOMO-1) (de Andrade et al., 2022; Sugimoto et al., 1992).

### The MDM2 inhibitor nutlin-3a enhances selinexor sensitivity in p53 wild-type-expressing sensitive cells

**Figure 5:**
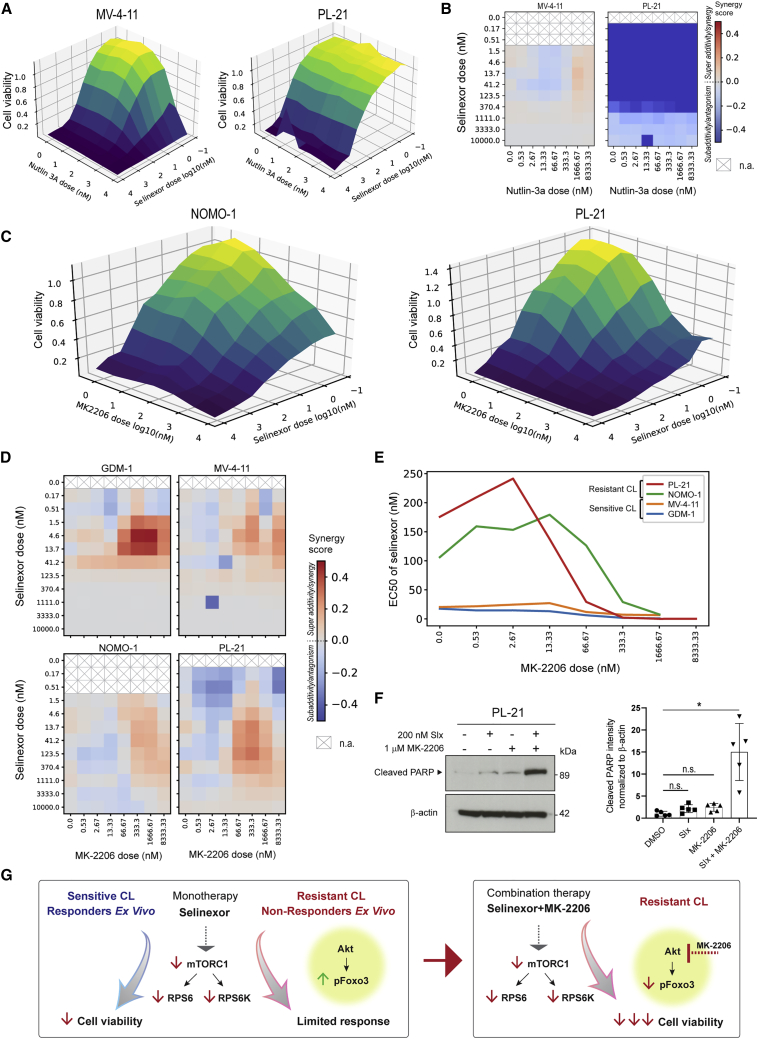
Selinexor combination therapy with MDM2 or AKT inhibition shows synergistic effects (A) 3D plots showing the mean relative cell viability (color scale, z axis) against selinexor and nutlin-3a doses (x and y axes) for AML cells treated with the drugs alone or in combination (n = 2–3 independent experiments; n = 4 technical replicates each dose). (B) Heatmaps showing the synergy scores derived for experiments in (A). (C) 3D plots showing mean relative cell viability (color scale, z axis) against selinexor and MK-2206 doses (x and y axes) for NOMO-1 and PL-21 cells treated with the drugs alone or in combination (n = 2–3 independent experiments; n = 4 technical replicates each dose). (D) Heatmaps showing the synergy scores for the different cell lines and drug combinations. (E) Calculated EC50 values for selinexor plotted for each dose of MK-2206 for each AML cell line as indicated. (F) Western blot (left) and quantification (right) of lysates from PL-21 cells treated with selinexor, MK-2206 alone, or the combination for 24 h and immunoblotted for cleaved PARP and β-actin (for reference). Quantification of blots (right) shows mean ± SD (n = 5). ^∗^p < 0.05 by two-sample Student’s t test with Bonferroni correction. n.s., non-significant. (G) Model of the selinexor response in *ex vivo* AML samples and cell lines. See also Figure S8.

Given the upregulation by selinexor and high functional score for p53 S315 in responders/sensitive cells, we hypothesized that functional p53 context might be required for selinexor sensitivity. Accordingly, we examined the benefit of combination with the MDM2 inhibitor nutlin-3a, which is known to stabilize and enhance p53 function (Lavin and Gueven, 2006). We found that only the sensitive cell lines with wild-type p53 expression (Figure 4D) responded to increasing doses of nutlin-3a with decreased cell viability after 72 h (Figure S8A), consistent with increased levels of p53 and its downstream effector p21 (Figure S8B). Moreover, in the selinexor-sensitive MV-4-11 cell line, combination treatment with selinexor demonstrated synergistic effect on cell viability 72 h post treatment at higher nutlin-3a concentrations (Figures 5A and 5B), in line with functional p53 being expressed at high levels, while there was no effect of the nutlin-3a combination in the p53 non-expressing PL-21 cells. Moreover, the synergistic effect in MV-4-11 cells was consistent with increased cleaved caspase-3 levels, which was also seen in the p53-expressing selinexor-sensitive GDM-1 cells (Figures S8C and S8D). These findings support the notion that functional p53 context provides for selinexor monotherapy sensitivity, which can be further enhanced with nutlin-3a.

### Selinexor in combination with the AKT inhibitor MK-2206 overcomes selinexor resistance

The observation of upregulated FOXO3 S253 phosphorylation by selinexor in non-responders/resistant cell lines suggests that dysregulated AKT signaling is a potential selinexor resistance mechanism. To test this, we assessed the combination of selinexor with the selective, allosteric AKT inhibitor MK-2206 (Hirai et al., 2010; Klaeger et al., 2017; Wiechmann et al., 2021). Sensitive and resistant AML cell lines were treated with increasing doses of selinexor alone or in combination with MK-2206, followed by cell viability measurements after 72 h (Figures 5C and S8E). As expected, the combination treatment with MK-2206 led to inhibition of phosphorylation of FOXO3 S253 and also confirmed AKT inhibition in GDM-1 and PL-21 cells (Figures S8F and S8G). Drug synergy scores were computed (Figure 5D) and all cell lines generally showed good synergy for selinexor doses ranging from 4.6 nM to 41.2 nM with MK-2206 doses between 333.3 and 1,666.6 nM. EC50 of selinexor for the different MK-2206 doses tested was computed for all four AML cell lines (Figure 5E). As suggested by the synergy scores, selinexor potency in resistant cell lines reached that of sensitive cells for MK-2206 dose levels of 333.3 nM and higher. Consistent with these effects, the combination of selinexor and MK-2206 resulted in increased PARP cleavage compared with single-agent treatments in the selinexor-resistant PL-21 cells (Figure 5F). These findings demonstrate the ability of AKT inhibition in combination with selinexor to overcome FOXO3-mediated selinexor resistance (Figure 5G).

### Spatial proteomics confirms nuclear FOXO3 translocation in PL-21 cells treated with selinexor in combination with MK-2206

**Figure 6:**
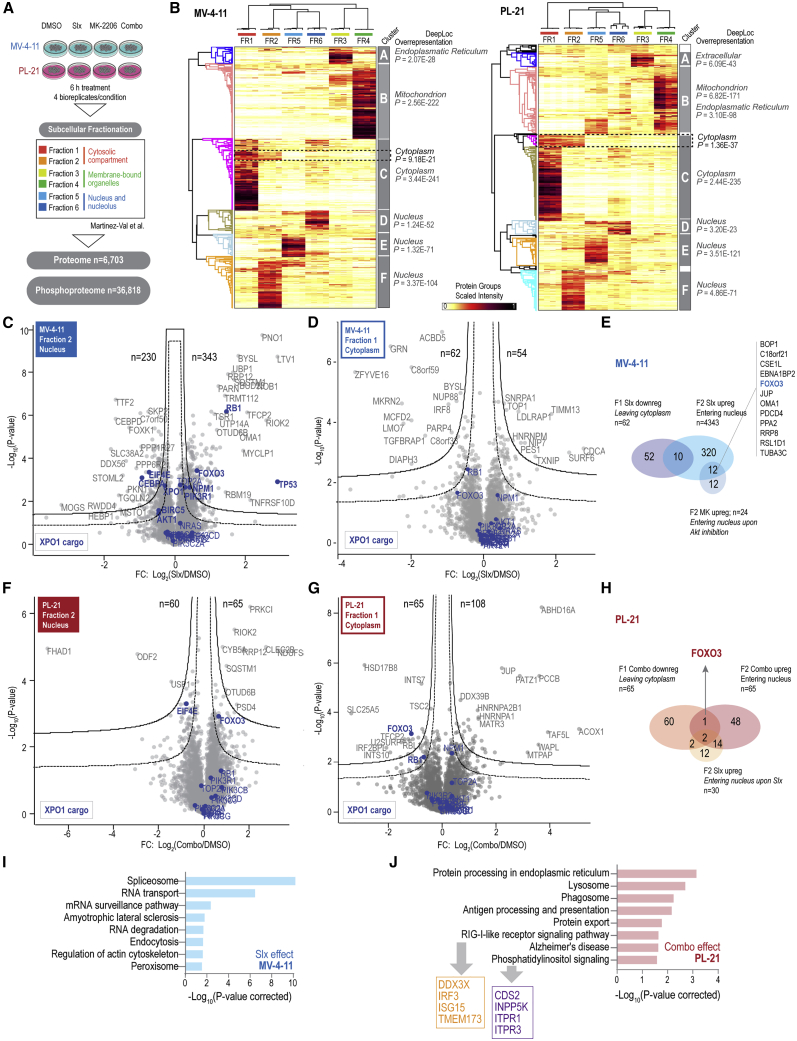
Subcellular proteome analysis of selinexor synergy combination with MK-2206 confirms FOXO3 nuclear translocation in PL-21 cells (A) Brief overview of the spatial-proteomics workflow and summary of results. (B) Heatmap of scaled intensities per fraction of the subcellular proteome of MV-4-11 (left) and PL-21 (right) cells (DMSO-treated cells; n = 4 independent experiments), showing protein and sample clustering. (C and D) Volcano plots showing differentially regulated proteins in the subcellular proteome analysis of fraction 2 (C) and 1 (D) of MV-4-11 cells. Fold change represents selinexor treatment versus DMSO control. Significance was deemed by a two-sided t test (FDR < 0.05 [dotted line], FDR < 0.01 [solid line], s0 = 0.1) using Perseus software. (E) Overlap of proteins identified in MV-4-11 cells to be significantly downregulated in fraction 1 and upregulated in fraction 2 by selinexor and upregulated by MK-2206 treatment in fraction 2. (F and G) Volcano plots showing differentially regulated proteins in the subcellular proteome analysis of fraction 2 (F) and 1 (G) of PL-21 cells. Fold change represents the combination treatment (selinexor and MK-2206) versus DMSO control. Significance was deemed by a two-sided t test (FDR < 0.05 [dotted line], FDR < 0.01 [solid line], s0 = 0.1) using Perseus software. (H) Overlap of proteins identified in PL-21 cells to be significantly downregulated in fraction 1 and upregulated in fraction 2 by the synergy combination and upregulated by selinexor in fraction 2. (I and J) KEGG pathway enrichment analysis for proteins significantly upregulated in fraction 2 by selinexor versus DMSO in MV-4-11 cells (I) and the synergy combination versus DMSO in PL-21 cells (J). See also Figures S9–S11; Tables S5 and S6.

Given that selinexor inhibits XPO1 and consequently affects the nucleocytoplasmic shuttling of proteins including FOXO3 upon dephosphorylation of S253, we applied a recent in-house-developed spatial proteomics workflow for studying subcellular phospho-signaling dynamics (Martinez-Val et al., 2021). We profiled six different subcellular fractions representing the cellular compartments of the cytosol, membrane-bound organelles and nucleus in MV-4-11 and PL-21 cells after treatment with DMSO, selinexor, MK-2206, or the combination quantifying 6,703 proteins and 36,818 confidently localized phosphorylation sites (class I; Olsen et al., 2006) (Figures 6A and S9A–S9E; Tables S5 and S6). Hierarchical clustering analysis of the scaled fractional protein and phosphorylation site intensities in the DMSO treatment group showed well-defined clusters (Figures 6B and S9F) that captured a distinct part of the cellular (phospho)proteome of MV-4-11 and PL-21 cells, as indicated by overrepresentation of the indicated ML-based DeepLoc terms (Almagro Armenteros et al., 2017). We tracked the dynamics of a selection of known XPO1 cargo proteins (Wang and Liu, 2019) and found, consistent with our western blotting experiments, that p53 was only detected in MV-4-11 cells (Figure S10A). It showed the highest intensity increase upon selinexor treatment in fraction 2 (DeepLoc: nuclear) supporting translocation to the nuclear compartment (Figure 6B). Moreover, global analysis of the selinexor-induced changes in fraction 2 (Figure 6C) showed a significant increase of well-known XPO1 cargo proteins, such as RB1, NPM1, PIK3R1, and FOXO3, in the sensitive MV-4-11 cells, with a concomitant trend for cytoplasmic depletion of FOXO3 and RB1 (Figures 6D and 6E). In contrast, no significant change was observed for these proteins in resistant PL-21 cells (Figure S10B). However, upon combination with MK-2206, FOXO3 showed translocation to the nucleus with concomitant cytoplasmic depletion in the resistant PL-21 cells (Figures 6F–6H and S10C). Moreover, MK-2206 treatment alone showed FOXO3 accumulation in the nucleus of MV-4-11 cells and not PL-21 (Figures 6E, S10D, and S10E), suggesting that selinexor is required for the accumulation of FOXO3 in the nuclear compartment in resistant cells. KEGG terms related to spliceosome and RNA transport were significantly overrepresented among the proteins translocating to the nucleus in MV-4-11 cells upon selinexor treatment (Figure 6I). By contrast, terms related to protein processing in the endoplasmic reticulum (ER), lysosome, and phagosome characterized the combination effect of proteins translocating to the nucleus in PL-21 cells, including pathways related to RIG-I-like receptor and phosphatidylinositol signaling, with the latter suggesting AKT inhibition to facilitate the translocation of components of its own pathway to the nucleus (Figure 6J).

In the subcellular phosphoproteome, differential phosphorylation site analysis of the selinexor response in MV-4-11 cells showed upregulation of phosphorylated ATM/ATR substrates, while p70S6K/mTOR substrates and key activation sites on MAPK1/3 (ERK) were downregulated in the cytoplasmic compartment by selinexor (Figure S11A). Sequence motif analysis showed an overrepresentation of Q in +1 of the phosphorylation site, supporting active DNA-damage signaling by ATM/ATR among the selinexor-upregulated sites. Moreover, proline-directed phosphorylation sites with P in +1 were overrepresented among selinexor-downregulated sites and aligned well with the observed reduced activity of MAPK1/3 in the cytoplasmic compartment (Figures S11A and S11B). Of note, consistent with the downregulation of p70S6K/mTOR substrates, we observed a significant upregulation of PIK3R1 in fraction 2 of the proteome data and a trend for upregulation of phosphoinositide-dependent kinase 1 (PDPK1) in fraction 6 (DeepLoc: nucleus) (Figure 6C; Table S5; fraction 6). This suggests that limited AKT activation can take place in the cytoplasm due to spatial constraint of AKT upstream kinases to the nuclear compartment. Relating this to the *ex vivo* model, we find that, among the top five highest functional scoring and downregulated phosphorylation sites (Figure 2A) for responders, the regulation of four sites fit well with our observations. First, both sites on Jun (S63 and S73; known MAPK3/ERK1 and CDK3 sites) (PhosphoSitePlus; Hornbeck et al., 2015) are downregulated and can be explained by the observed MAPK1/3 inhibition (Figure S11A) in MV-4-11 cells. Second, the MAF1 site (S68) comprises a direct site phosphorylated by mTOR (Michels et al., 2010), supporting our findings that sites on mTOR signaling proteins are significantly downregulated (Figure S11A). Finally, PRKCQ T538 (in the kinase activation loop) is phosphorylated by PDPK1 (Sparatore et al., 2003), and hence potentially downregulated as a consequence of PDPK1 nuclear retention.

In contrast, for resistant PL-21 cells, we observed that only synergistic treatment combination resulted in regulation of ATM/ATR and p70S6K substrates, and mTOR signaling mimicking the selinexor single-treatment response in MV-4-11 cells (Figures S11A, S11C, and S11D). For PL-21 cells, AKT2 was significantly increased in nuclear fraction 5 upon combination treatment, suggesting a dual mechanism for the downregulation of p70S6K substrates/mTOR signaling, including the inhibition of AKT by MK-2206 and the nuclear retention of AKT2 away from its downstream targets (Table S5; fraction 5). Interestingly, the mTOR S2454 autophosphorylation site was upregulated by selinexor monotherapy, while it was downregulated together with mTOR S2448 (a known AKT/p70S6K target site) by the drug combination, supporting the model proposed in Figure 5G. Finally, in fraction 2, representing the nuclear compartment, we detected multiple upregulated phosphorylation sites on p53 and FOXO3 for MV-4-11 upon selinexor single treatment, while the upregulation of multiple FOXO3 phosphorylation sites was only seen in PL-21 cells upon treatment with the drug combination (Figures S11E and S11F).

## Discussion

There is a significant need for improved treatment options for AML. The results presented in the current study uncover several clinically actionable, molecular contexts predicting sensitivity to both single-agent selinexor as well as to rational combination therapy with selinexor in a subset of AML, in particular the M1 and M4 subgroups, not deducible through genetic characterization. First, we clearly demonstrate that activated AKT-FOXO3 signaling is a key resistance mechanism to selinexor, which can be overcome by combining selinexor with the AKT inhibitor MK-2206. Our findings also show that functional p53 tumor suppressor activity is a requirement for selinexor single-agent activity in AML and that combination therapy with the MDM2 inhibitor nutlin-3a leads to elevated p53 levels and synergistic cell killing. These findings support additional testable hypotheses that combinations with other MDM2 inhibitors or inhibitors of MYC signaling, such as a bromodomain and extra-terminal motif inhibitor (BETi), might synergize with selinexor in AML and other tumor types with functional p53 through alleviation of suppression of p53 activity. Despite a limited sample size, our phosphoproteome analysis covers a significant fraction of intermediate and high-risk patients with unmet therapeutic needs. Our functional approach is inherently agnostic to underlying genetic alterations and hence offers an alternative approach for rational therapeutic intervention to improve patient outcomes. It remains to be seen in larger cohorts whether the findings of this study associate with specific AML subgroups and specific genetic abnormalities in AML.

A key finding in the current study is the significant selinexor-induced upregulation of p53 S315 phosphorylation in responder AML patient samples and the sensitive AML cell lines (Figures 2A and 4A). This phosphorylated site binds the prolyl isomerase PIN1 in the nucleus, leading to transcriptional activation of wild-type p53 (Wulf et al., 2002; Zacchi et al., 2002; Zheng et al., 2002). Consistent with this, a p53 S315A mutant allele is less transcriptionally active than wild-type p53 (Ito et al., 2001). The significance of this finding is further substantiated by a concomitant upregulated phosphorylation of S166 in MDM2, a negative regulator of p53, in responder AML patient samples. A majority of AML cases contain wild-type *TP53*, and only 8% of *de novo* AML cases contain *TP53* mutations according to TCGA. However, p53 is often rendered functionally deficient by over-expression of MDM2 (Latif et al., 2021). Selinexor blocks nuclear export and traps both proteins in the nucleus, thus hindering the MDM2-dependent shuttling of MDM2-p53 targeted for cytoplasmic degradation and hence induces transcriptional upregulation through phosphorylation of these sites (O'Keefe et al., 2003). These findings suggest that selinexor single-agent activity in AML is dependent, at least in part, on minimally one functional *TP53* allele (Nishida et al., 2019), as shown for other tumor types, including diffuse large B-cell lymphoma (DLBCL) and gastric cancer (Deng et al., 2020; Subhash et al., 2018).

Another major finding in this study is the acute upregulation of FOXO3 S253 phosphorylation in the non-responding subset of AML samples, consistent with dysregulated AKT signaling. This is clinically actionable, proposing that the selective, potent AKT inhibitor MK-2206 is a rational drug combination for treatment of AML to overcome selinexor resistance. The importance of the core PI3K-AKT-mTOR pathway in AML is underscored by its amplification in at least 60%–80% of AML patient blasts (Herschbein and Liesveld, 2018). Despite preclinical evidence supporting a beneficial effect of rapamycin and MK-2206 monotherapy in AML cells, clinical results have been disappointing (Boehm et al., 2009; Callera et al., 2008; Konopleva et al., 2014; Recher et al., 2005). This may relate to cellular pathway crosstalk, perturbation of negative feedback loops, as well as co-existing constitutive pathways supporting growth and survival (Herschbein and Liesveld, 2018). Moreover, subsets of AML patients display varying degrees of network plasticity defining *in vivo* susceptibility to PI3K-AKT-mTOR inhibition, which has been shown to be most effective in patients with low levels of constitutive signaling (Nepstad et al., 2018). The mTOR protein functions as part of two major protein complexes; mTORC1 and mTORC2. The mTORC1 complex directly phosphorylates the RSK p70S6 kinase and the translational repressor 4E-BP1 involved in mRNA translation, resulting in cell growth, whereas mTORC2 directly phosphorylates AKT S473, resulting in its activation and resistance to apoptosis (Gentzler et al., 2012; Herschbein and Liesveld, 2018). Our data suggest a differential dependency on and central role of the PI3K-AKT-mTOR signaling axis in responder and non-responder AML blasts, resulting in a differential sensitivity to selinexor monotherapy. First, in sensitive cells, we observe that selinexor traps the AKT upstream kinases PIK3R1 and PDPK1 in the nucleus, thereby limiting their access to activation of cytoplasmic AKT, consistent with the observed downregulation of p70SK6/mTOR substrates and FOXO3 nuclear translocation. PIK3R1 and PDPK1 can localize to the nucleus and serve as XPO1 cargo (Davis et al., 2015; Sephton et al., 2009; Wang and Liu, 2019). Although this regulation by selinexor seems lost in the resistant cells, it offers the unique opportunity to exploit the observed acute selinexor-induced upregulated AKT signaling through mTOR (Figure S11C; upregulated mTOR_S2454 autophosphorylation site) and reflected in high level of FOXO3 S253 phosphorylation as shown in the *ex vivo* non-responders and the resistant PL-21 cells (Figures S2E and 3F). Importantly, we demonstrate that, when AKT signaling is targeted by MK-2206 in combination with selinexor, we observe nuclear translocation of FOXO3 (Figure 6F), which is not evident upon MK-2206 treatment alone (Figure S10E).

The success of new drug candidates and combination therapies depends on rational clinical trial design and proper patient selection. In this study, we have identified molecular context of p53 for selinexor single-agent activity in AML consistent with enhanced efficacy of selinexor in combination with the MDM2 inhibitor nutlin-3a. Moreover, we identify dysregulated AKT-FOXO3 signaling as a key resistance mechanism in selinexor-resistant cells and, accordingly, that the rational combination with MK-2206 overcomes this resistance, as shown in non-responders using *ex vivo*-treated patient-derived AML samples and in AML cell lines. Importantly, only a subset of non-responders showed increased AKT signaling and FOXO3 S253 phosphorylation, pinpointing the importance of predictive biomarker identification for patient responsiveness. Our data demonstrate a mechanistically comprehensive, functional basis for synergy of AKT inhibition with selinexor to overcome selinexor resistance that might be clinically relevant in a subset of AML patients. Future studies will illuminate how generalizable these findings are in the clinical setting.

In summary, our study provides a comprehensive characterization of the phosphoproteome changes associated with selinexor treatment in *ex vivo* AML blasts from patients and AML cell lines, including subcellular proteome dynamics for the latter. We demonstrate the potential of our PAFS approach to identify clinically actionable network nodes from the phosphoproteome data and target these in rational combinations to overcome drug resistances that are not possible to deduce through genetic analyses.

### Limitations of the study

In the current study, we highlight three main limitations. First, given the high degree of AML heterogeneity, the sample size of our current study presents insufficient power to uncover strong subtype-specific responses to treatment. Second, despite the known advantage of using TMT for peptide labeling due to its low index of missing values within a single TMT batch, the comparison across multiple batches is challenging. The analysis of larger cohorts including treatment conditions poses the risk of inflating the number of missing values as multiple batches are integrated. Third, our PAFS approach holds an inherent bias in assigning higher scores for well-studied phosphorylation sites. Thus, less well-studied phosphorylation sites may, despite lower functional scores, still be of key biological importance when regulated in any given setup.

Note that our findings of the therapeutically actionable enhancing effects of MDM2 and AKT inhibition with selinexor, have just been reported (PMID: 35785714 and PMID: 35668193, respectively).

## STAR★Methods

### Key resources table

**Table:** 

REAGENT or RESOURCE	SOURCE	IDENTIFIER
**Antibodies**		

Anti-β-Actin	Santa Cruz	Cat# sc-47778; RRID:AB_626632
Anti-phospho-Rb (S780) (D59B7)	Cell Signaling Technologies	Cat# 8180; RRID:AB_10950972
Anti-Rb (4H1)	Cell Signaling Technologies	Cat# 9309; RRID:AB_823629
Anti-cleaved PARP	Cell Signaling Technologies	Cat# 5625; RRID:AB_10699459
Anti-cleaved caspase 3	Cell Signaling Technologies	Cat# 9664; RRID:AB_2070042
Anti–phospho-AKT (S473)	Cell Signaling Technologies	Cat# 9271; RRID:AB_329825
Anti-AKT	Cell Signaling Technologies	Cat# 9272; RRID:AB_329827
Anti–phospho-FoxO3a (S253) (D18H8)	Cell Signaling Technologies	Cat# 13129; RRID:AB_2687495
Anti-FoxO3a (D19A7)	Cell Signaling Technologies	Cat# 12829; RRID:AB_2636990
Anti-p53	Cell Signaling Technologies	Cat# 48818; RRID:AB_2713958
Anti-p21	Cell Signaling Technologies	Cat# 2947; RRID:AB_823586
Anti-GAPDH	Abcam	Cat# Ab8245; RRID:AB_2107448

**Biological samples**		

Patient derived leukemic cells	Hôpital Saint-Louis – Cellulothèque Laboratorie Central d'Hématologie	N/A

**Chemicals, peptides, and recombinant proteins**		

Selinexor (KPT-330)	Karyopharm	N/A
MK-2206 AKT inhibitor	Selleck Chemicals	Cat# S1078
Nutlin-3a	MedChemExpress	Cat# HY-10029

**Critical commercial assays**		

TMT10plex isobaric label reagent set	Thermo Fischer Scientific	Cat# 90406
TMT11-131C Label Reagent	Thermo Fischer Scientific	Cat# A34807

**Deposited data**		

Phosphoproteomics data: raw MS data and associated tables	http://proteomecentral.proteomexchange.org	PXD017660, PXD033515 and PXD033527

**Experimental models: Cell lines**		

GDM-1	DSMZ	ACC 87
MV4-11	ATCC	CRL-9591
PL-21	DSMZ	ACC 536
NOMO-1	DSMZ	ACC 542
SKM-1	DSMZ	ACC 547
THP-1	DSMZ	ACC 16
KG-1	DSMZ	ACC-14
MOLM-13	DSMZ	ACC-13

**Software and algorithms**		

MaxQuant v. 1.6.0.17	Cox and Mann, 2008	https://www.maxquant.org/
Spectronaut v15.6/v15.7	Bruderer et al., 2017	https://biognosys.com/software/spectronaut/
WebLogo	Crooks et al., 2004	https://weblogo.berkeley.edu/logo.cgi
IceLogo	Colaert et al., 2009	https://iomics.ugent.be/icelogoserver/
Perseus v. 1.6.2.2	Tyanova et al., 2016	https://www.maxquant.org/perseus/
Bioinformatic analysis pipeline	This manuscript	https://doi.org/10.5281/zenodo.6838174
SciPy v1.4.1	Virtanen et al., 2020	https://www.scipy.org/
VSN v3.54.0	Huber et al., 2002	http://bioconductor.org/packages/vsn/
Limma v3.42.2	Ritchie et al., 2015	https://bioconductor.org/packages/limma/
Kinact v1.0	Wirbel et al., 2018	https://github.com/saezlab/kinact
Piano v2.2.0	Varemo et al., 2013	https://bioconductor.org/packages/piano/

**Other**		

MSigDB	Subramanian et al., 2005	http://www.gsea-msigdb.org/gsea/msigdb/index.jsp
OmniPath	Turei et al., 2021	https://omnipathdb.org/

### Resource availability

#### Lead contact

Further information and requests for resources and data should be directed to and will be fulfilled by the lead contact, Jesper Velgaard Olsen (jesper.olsen@cpr.ku.dk).

#### Materials availability

This study did not generate new unique reagents.

### Experimental model and subject details

#### Patient information and ethics

Primary patient AML blasts were collected from bone marrow aspirates or peripheral blood samples after obtaining patient written informed consent under Saint-Louis Hospital Internal Review Board-approved protocols. These studies were conducted in accordance with recognized ethical guidelines from the Declaration of Helsinki and were approved by an institutional review board. The *ex vivo* AML model included 44 patients in the age range 20–84 years (21 males, 23 females) with the characteristics as described in Table S1.

#### *Ex vivo* AML cell culture

Samples were quickly thawed by swirling the frozen vial in a 37°C water bath. Cells were transferred to a sterile 15 mL falcon tube followed by addition of 10 mL of RPMI 1640 medium (Corning, Glutagro) supplemented with 10% fetal bovine serum (Gibco) and penicillin-streptomycin (100 units/mL and 100 μg/mL, HyClone, GE Healthcare). Cells were centrifuged at 300G for 10 min. The supernatant was removed and the tube was gently tapped to loosen the pellet. Cells were then gently resuspended in 900 μL medium followed by dropwise addition of 100 μL of 1 mg/mL DNase (STEMCELL Technologies) for a final concentration of 0.1 mg/mL DNase. Cells were incubated with DNase at RT for 15 min. Treatment was stopped by adding 10 mL of medium followed by centrifugation as above. Cells were resuspended in medium supplemented with the following growth factors and cytokines; TPO 50 ng/mL (Peprotech), Flt-3 50 ng/mL (ORF Genetics, Iceland), SCF 25 ng/mL (ORF Genetics), IL-3 10 ng/mL (ORF Genetics), IL-6 10 ng/mL (Peprotech), GM-CSF 10 ng/mL (ORF Genetics), G-CSF 10 ng/mL (ORF Genetics) EPO 10 ng/mL (Peprotech). Total number and percentage of viable cells was determined using trypan blue staining and an automated cell counter (Countess™, Invitrogen). Primary cells were immediately used in subsequent experiments.

#### AML cell lines

The human AML cell lines GDM-1, PL-21, NOMO-1, MV-4-11, SKM-1, THP-1, KG-1, and MOLM-13 were obtained from DSMZ and cultured in RPMI 1640 including 2 mM L-glutamine (Corning), supplemented with 10% fetal bovine serum (Gibco) and penicillin-streptomycin (100 units/mL and 100 μg/mL; Gibco). Cell lines were maintained at 37°C in a humidified atmosphere at 5% CO_2_.

### Method details

#### Reagents

Selinexor (KPT-330) was provided by Karyopharm Therapeutics Inc. and MK-2206 was purchased from Selleck Chemicals. Nutlin-3a was purchased from MedChemExpress. Compounds were dissolved in DMSO, and the final concentration of DMSO was kept at maximum 0.02% (v/v) during cell culture treatment.

#### Cell viability assay

Cells were seeded in 384 well plates at a density of 5000 cells per well in 25 μL medium. Drug treatment was performed 2 hours post-thawing by adding increasing doses of drug from a 3-fold dilution series to test 11 different concentrations. Each dose was added in four replicate wells, and the plate was incubated at 37°C. Following selinexor treatment, cell viability was assessed 48 h post-drug treatment by adding PrestoBlue reagent (ThermoFisher) and measuring fluorescence using a SpectraMax i3 multi-mode plate reader (Molecular Devices). Following nutlin-3a treatment, cell viability was assessed 72 h post treatment by adding Cell Titer Glo 2.0 (Promega) and measuring luminescence in a SpectraMax i3 multi-mode plate reader. For drug combinations of selinexor and MK-2206, or selinexor and nutlin-3a, cells were seeded in 384-well plates followed by drug treatment using a 3-fold serial dilution of selinexor and a 5-fold serial dilution of either MK-2206 or nutlin-3a. Cell viability was assessed 72 h post drug treatment by adding Cell Titer Glo 2.0 (Promega) and measuring luminescence in a SpectraMax i3 multi-mode plate reader.

#### Dose-response models

To determine the drug response of the different samples, we first transformed the survival data of each replicate to their reference sample (i.e., response at drug concentration 0 nM) then computed the mean response across the four replicates for each drug concentration and sample. The drug concentration and mean response were fitted to a modified Hill function describing the dose response:

(Equation 1) $$R=m\frac{D^{n}}{k^{n}+D^{n}}$$

where R is the relative response (dimensionless, between zero and one) and D is the drug dose (in nM). The parameters m and n (dimensionless) control the intercept and steepness of the curve respectively, k (nM) also contributes to the curvature of the function. The models were fitted using the nonlinear least squares method implemented in the scipy Python package (Virtanen et al., 2020) (v1.4.1). Considering our data and feasibility, we considered a parameter initial guess of 1000 nM for k, 1 for m and −1 for n. Furthermore, the following boundaries were also considered in the optimization: [0, ∞) for k and m and (-∞, 0] for n. Once the model parameters were fitted, the EC50 was computed by considering R = 0.5 and solving (1) for D (which will correspond to EC50), such that:

(Equation 2) $$EC_{50}=\sqrt[{n}]{\frac{k^{n}}{2m-1}}$$

#### Western blotting

After drug treatment, cells were collected and washed in PBS, lysed in RIPA buffer containing protease and phosphatase inhibitors while standing on ice for 20 min with vortexing every 5 min. Lysates were centrifuged, collected, and boiled for 10 min at 70°C together with LDS sample buffer (NuPAGE, Invitrogen) and reducing agent (NuPAGE, Invitrogen). Samples were separated on NuPAGE 4–12% Bis-Tris gels (Invitrogen) and transferred to low-fluorescent PVDF membranes (Immobilon-FL, Merck Millipore) using a Power Blotter (Invitrogen). Membranes were blocked in blocking buffer (Blocker™ FL, Thermo Fisher), incubated with primary antibodies over night at 4°C, washed 3 × 5 min in PBS-Tween (0.05%), incubated with species-specific secondary antibody for 1 h at RT (Alexa Fluor™ Plus 647 or Alexa Fluor™ Plus 800, Invitrogen) or species-specific peroxidase-conjugated secondary antibodies, washed 3 × 5 min, and developed using an iBright™ FL-1500 (Thermo Fisher) or the Novex ECL Chemiluminescent Substrate Reagent Kit (Invitrogen) and band detection by exposure to Hyperfilm (Amersham, GE Healthcare). Signal intensity was quantified using iBright Analysis Software (Thermo Fisher) or ImageJ. Primary antibodies used were anti rabbit-phospho-Rb (S780) (D59B7) (CST8180) and mouse anti-Rb (4H1) (CST9309), rabbit anti-cleaved PARP (CST5625), rabbit anti-cleaved caspase 3 (CST9664) mouse anti-β-actin (sc47778), rabbit anti–phospho-AKT (S473) (CST9271) and anti-AKT (CST9272), rabbit anti–phospho-FoxO3a (S253) (D18H8) (CST13129) and anti-FoxO3a (D19A7) (CST12829), mouse anti-p53 (CST48818), rabbit anti-p21 (CST2947), mouse anti-GAPDH (Ab8245; Abcam).

#### Selinexor treatment of cells for phosphoproteomics

Cells were seeded in 6-well plates and subsequently treated 2 hours post-thawing with either 1 μM selinexor or DMSO for 6 h. Primary cells were seeded in medium supplemented with growth factors and cytokines as specified above. For cell lines, a minimum of 20 million cells were seeded per treatment and biological replicates. For primary cells, varying numbers of viable cells obtained from each vial prevented us from seeding the same number of viable cells from each sample. Thus, numbers of seeded primary cells varied between 3 million up to >20 million.

#### Sample preparation for TMT labelling and phosphoproteomics

Cells were washed in PBS 6 hours post-treatment (1 μM selinexor or 0.01% DMSO) and lysed for 10 min at 99°C in 6 M guanidine-HCl, 100 mM Tris pH 8.5, 5 mM TCEP and 10 mM CAA and whole cell extracts were sonicated. Proteolytic digest was performed by Lys-C (Wako) in an enzyme/protein ratio of 1:300 (w/w) for 1 hour, followed by a dilution with 25 mM Tris buffer pH 8.5, to 2 M guanidine-HCl and further digested overnight with trypsin (Sigma-Aldrich) 1:100 (w/w). Protease activity was quenched by acidification with TFA and the resulting peptide mixture was concentrated on C18 Sep-Pak (Waters). Peptides were eluted with 40% ACN, followed by 60% ACN and the ACN was subsequently evaporated using a SpeedVac vacuum concentrator. The final peptide concentration for each sample was estimated by measuring absorbance at A280 on a NanoDrop (Thermo Scientific). The samples were assigned to one of four TMT-11 multiplexing label sets, including a pooled mix of peptides from different patient AML samples serving as a common reference between TMT sets. Equal amounts of peptide from each sample (*Ex vivo* samples; TMT set 1: 100 μg/sample, TMT set 2: 76 μg/sample, TMT set 3: 67 μg/sample, TMT set 4: 51 μg/sample, and for cell lines; 300 μg/sample) were labeled with one of eleven (*ex vivo*) or eight (cell lines) different TMT-reagents according to the manufacturer’s protocol (ThermoScientific). After labeling, the samples were pooled and adjusted to 88% ACN, 6% TFA and phosphopeptides were enriched by two sequential rounds using titanium dioxide beads (TiO2; GL Sciences). TiO2 beads were pre-incubated in 2,5-dihydroxybenzoic acid (20 mg/mL; Sigma-Aldrich) in 80% ACN/1% TFA (5 μL/mg of beads) for 20 min. Beads equivalent to 0.5× starting protein amount were added to each pooled TMT multiplexed sample set, which were then incubated for 20 min while rotating. Beads were washed with 80% ACN/6% TFA, 50% ACN/6% TFA, 80% ACN/1% TFA, 50% ACN/1% TFA and 10%/1% TFA and transferred to C8 StageTips. Phosphopeptides are then eluted with 5% ammonia (Merck) and 10% ammonia/25% ACN and concentrated in a SpeedVac and fractionated with high-pH-reversed-phase fractionation into 12 fractions. Moreover, for cell lines an aliquot of eluate from each round of enrichment was used for single shot MS analysis.

#### Sample preparation for single-shot proteome analysis

Cells from PL-21, NOMO-1, GDM-1 and MV-4-11 were cultured for 3 days and collected was washed twice with PBS. The cell pellets were lysed for 10 min at 95°C in 100 mM Tris-HCl (pH 8.5), 5% SDS, 5 mM TCEP, and 10 mM CAA, followed by the sonication. Samples were centrifuged at 4000 g for 10 min, and the supernatants collected. 100 μg of proteins were digested overnight 37°C using PAC method (Batth et al., 2019) in 50 mM triethylammonium bicarbonate (TEAB) containing 0.2 μg of Lys-C and 0.4 μg of Trypsin. The digest was quenched by acidification with formic acid (FA). 750 ng of peptides were loaded on to Evotips (Evosep) for proteome analysis.

#### Subcellular fractionation and LC-MS/MS analysis

Cells were seeded in 15 cm dishes at a density of 15 million (PL-21) or 20 million (MV-4-11) cells/dish, and incubated for 6 h with 1 μM selinexor, 1 μM MK-2206, both inhibitors, or DMSO (each condition repeated in four biological replicates). After the incubation with the inhibitors, subcellular fractionation into six fractions and sample preparation for MS analysis were performed as previously described (Martinez-Val et al., 2021), with slight modifications. The fractions were incubated for 10 min at 90°C with 0.3% SDS, 5 mM TCEP, and 10 mM CAA, followed by the digestion using PAC method (Batth et al., 2019) and incubated overnight at 37°C in 50 mM TEAB containing 0.5 μg of Lys-C and 1 μg of Trypsin. After the digestion, the beads were removed and the solution was acidified with 40 μL of 10% formic acid (FA). 20 μL of each sample were loaded to Evotips (Evosep) for proteome analysis. The remaining solution were loaded to Sep-Pak tC18 96-well μElution Plate (5 mg Sorbent per well, 37–55 μm, Waters) for desaltning and eluted with 30 μL of 40% ACN and 60% ACN subsequently. The eluents were pooled and subjected to phosphopeptide enrichment. The eluents were adjusted to 80% ACN, 5% TFA, and 1M glycolic acid (GA) and incubated with 20 μL (20 mg/mL) of TiO_2_ beads (MagResyn), followed by the subsequent wash with 80% ACN/5% TFA/1 M GA, 80% ACN/1% TFA, and 10%ACN/0.2% TFA. The enriched peptides were eluted in 200 μL of 1% ammonia, acidified with 50 μL of 10% TFA, filtered, and loaded on to Evotips for phosphoproteome analysis.

#### LC–MS/MS and MS data analysis

For TMT-based phosphoproteomics, peptides were analyzed using online nanoflow LC-MS/MS on a Q Exactive HF-X mass spectrometer (Thermo Fisher Scientific), which was interfaced with an EASY-nLC system (Proxeon, Odense, Denmark) equipped with a nanoelectrospray ion source. Raw MS files were analyzed by MaxQuant software version 1.6.0.17 using the Andromeda search engine (Cox and Mann, 2008). Proteins were identified by searching the HCD-MS/MS peak lists against a target/decoy version of the human UniProt protein database (UP000005640 release 2017_04 with 21,054 reviewed entries) using default settings. Carbamidomethylation of cysteine was specified as fixed modification and protein N-terminal acetylation, oxidation of methionine, pyro-glutamate formation from glutamine and phosphorylation of serine, threonine and tyrosine residues were considered as variable modifications. Minimum peptide length was 7 amino acids, “maximum peptide mass” was set to 7,500 Da, the “modified peptide minimum score” and “modified maximum peptide score” were set to 25. Label min. ratio count set to 1. Peptide spectrum match (PSM), protein, and site FDR was set to 0.01. Everything else was set to default values.

Samples for single-shot proteome analysis and spatial proteome analysis of subcellular fractions were analyzed on the Evosep One system using an in-house packed 15 cm, 150 μm i.d. capillary column with 1.9 μm Reprosil-Pur C18 beads (Dr. Maisch, Ammerbuch, Germany) using the pre-programmed gradient for 30 samples per day or 60 samples per day (phospho only). Column temperature was set to 60°C using an integrated column oven (PRSO-V1, Sonation, Biberach, Germany) and interfaced online with the Orbitrap Exploris 480 MS (Thermo Fisher Scientific, Bremen, Germany) using Xcalibur (tune version 1.1). Spray voltage was set to 2 kV, funnel RF level at 40, and heated capillary temperature at 275°C. For single-shot and full-proteome analysis of subcellular fractions in data-independent acquisition (DIA) mode, full MS resolutions were set to 120,000 at m/z 200 and full MS AGC target was 300% with an IT of 45 ms. Mass range was set to 350–1400. AGC target value for fragment spectra was set at 100%. 49 windows of 13.7 m/z scanning from 361 to 1033 m/z were used with an overlap of 1 Da. Resolution was set to 15,000 and IT to 22 ms and normalized collision energy was 27%. For phosphoproteome analysis using DIA, we employed 17 windows of 39.5 m/z scanning from 472 to 1143 m/z with 1 m/z overlap. Resolution was set to 45,000 and IT to 86 ms. Normalized collision energy was set at 27%. All data were acquired in profile mode using positive polarity.

All samples acquired in DIA-mode were analysed by Spectronaut v15.6 (spatial proteomics) or v15.7 (single-shot proteomes) with a library-free approach (directDIA) using the human database (UP000005640_9606 release 2018 with signal peptide removed containing 21,007 reviewed entries), supplemented with a database of common contaminants (Bruderer et al., 2017). For proteome analysis, carbamidomethylation of cysteine was specified as fixed modification and protein N-terminal acetylation and oxidation of methionine, were considered as variable modifications. Additionally for phosphoproteome analysis, phosphorylation of serine, threonine and tyrosine were included as well. The maximum number of variable modifications per peptide was limited to 3. Peptide spectrum match (PSM), pepetide and protein group FDR was set to 0.01. Cross-run normalization was turned off for spatial proteomics searches and for protein quantification, major protein group aggregation method was changed to sum.

#### Bioinformatics analysis of phosphoproteomics data

##### Data normalization

All phosphosite intensities were normalized with the Variance Stabilization Normalization (VSN) method by using the vsn package in R v3.54.0 (Huber et al., 2002). The method was applied separately on each TMT batch of samples and then batch effects were removed with the limma package v.3.42.2 (Ritchie et al., 2015).

Only peptides with a phosphorylation site localization probability of at least 0.75 (class 1) were included in the bioinformatic analyses. Volcano plots showing differential phosphorylation upon selinexor treatment for *ex vivo* and cell lines samples were generated by plotting the –log_10_ transformed and FDR-adjusted p values (FDR <0.05) derived from a two-sided t-test versus log_2_-transformed fold changes as indicated. Statistical significance was determined on the basis of a hyperbolic curve threshold of s0 = 0.1 derived from statistical analysis using microarrays (SAM testing; described in (Tusher et al., 2001) and for phosphoproteomics analysis in (Hogrebe et al., 2018)) using Perseus version 1.6.2.2. To assess sequence bias around regulated phosphorylation sites sequence motif, logo plots (±6 amino acids adjacent to the identified phosphorylated sites) were generated and visualized using the WebLogo tool (Crooks et al., 2004) with default parameters (p < 0.01). From a list of 120.000 phosphorylation sites, we integrated phosphosite-specific functional scores as reported by Ochoa et al. (Ochoa et al., 2020) to rank and prioritize the significantly regulated phosphorylation sites in our analysis.

##### Gene set enrichment analysis (GSEA)

Gene Set Enrichment Analysis was performed using the piano package v2.2.0 (Varemo et al., 2013) in R based on the t- and p-values obtained from the differential expression analysis. The gene sets were obtained from the canonical pathways defined by MsigDB v7.2 (Subramanian et al., 2005). This package allowed us to compute the enrichment rankings by using different statistical methods and combine their results based on the median ranks of each method (i.e. consensus score). The methods used in this case are: stouffer, reporter, tailStrength, wilcoxon, mean, median, sum, and page (see Väremo et al. for more information). The enriched gene sets are classified into different directionality classes according to the directions in the regulation of their members. These are distinct-directional - for gene sets whose members have a clear up- or downregulation -, mixed-directional - for gene sets whose members have a major regulation direction but some point towards the opposite -, and the non-directional class - for gene-sets with no clear distinction on the directionality of its members. In this study, only the distinct-directional gene sets have been considered.

##### Kinase-substrate enrichment analysis (KSEA)

Kinase-Substrate Enrichment Analysis was computed from the log2(FC) values using the kinact v1.0 (Wirbel et al., 2018) package in Python. The kinase-substrate (i.e. kinase-phosphosites) associations were downloaded from OmniPath (Turei et al., 2021) (as of November 17^th^ 2020). The algorithm implements the enrichment method as described by Casado et al. which infers kinase activities based on the relationships between them and the measured phosphosites (Casado et al., 2013).

##### Drug combination synergy scoring

Based on the dose-response model shown in Equation (1) and given two drugs *a* and *b,* we derive the theoretical dose of *b* that achieves the same response of *a* as:

(Equation 3) $$R_{a}\left(a\right)=R_{b}\left(b\right)=\frac{m_{a}a^{p}}{k_{a}^{p}+a^{p}}=\frac{m_{b}b^{q}}{k_{b}^{q}+b^{q}}$$

(Equation 4) $$\hat{b}\left(a\right)=\sqrt[{q}]{\frac{m_{a}a^{p}k_{b}^{q}}{k_{a}^{p}m_{b}-m_{a}a^{p}+m_{b}a^{p}}}$$

Then, considering Loewe additivity principle, the expected response of any combination of *a* and *b* is defined by:

(Equation 5) $$\hat{R_{ab}}\left(a,b\right)=R_{b}\left(b+\hat{b}\left(a\right)\right)$$

The synergy score of a drug combination is then computed as the difference between the expected response and the actual measured response:

$$S_{ab}=\hat{R_{ab}}-R_{ab}$$

Such that negative scores denote subadditivity (antagonistic effect) and a positive score shows superadditivity (synergistic effect).

#### Bioinformatics of DIA-data sets

Dataset normalization and imputation of single-shot proteomes were performed using the Spectronaunt software with enabled cross run normalization and global imputation. For the subcellular proteome and phosphoproteome, each fraction was individually normalized and imputed using Prostar software (Wieczorek et al., 2017). The data set were filtered requiring at least 4 (proteome) or 3 (phosphoproteome) values for at least one condition. Global quantile alignment was used for normalization. For missing values imputation, slsa and det quantile algorithms were used partially observed values (POV) and missing on entire condition (MEC), respectively. Volcano plots showing differential regulation were generated using Perseus as described in the previous section. KEGG term overrepresentation was calculated by InnateDB (Breuer et al., 2013) with default settings for pathway overrepresentation analysis. To asses for sequence bias around the regulated phosphorylation sites, sequence motif logo plots (±6 amino acids adjacent to the identified phosphorylated sites) were generated and visualized using the IceLogo software (Colaert et al., 2009) with default parameters (p < 0.05).

### Quantification and statistical analysis

#### Statistical analysis

Statistical analysis of MS data is described in the Bioinformatic analysis sections. Statistical tests of Western blot quantifications were applied for experiments performed in three or more biological replicates as indicated in the figure legends. Results shown are the mean of measurements ±SD. p values were calculated using a two-sample Student’s t test and Bonferroni correction was used to correct for multiple t test comparisons. Significance was concluded when p < 0.05 indicated by ^∗^ in the figures.

## Data Availability

• The raw MS data and associated tables have been deposited to the ProteomeXchange Consortium (http://proteomecentral.proteomexchange.org) via the PRIDE partner repository and are publically available as of the date of publication. Accession numbers are listed in the key resources table. • Code for bioinformatics analysis of phosphoproteomics data is publically available as of the date of publication at GitHub repository: https://github.com/saezlab/OncoSignature and archived at Zenodo with the DOI: https://doi.org/10.5281/zenodo.6838174. • Any additional information required to reanalyze the data reported in this paper is available from the lead contact upon request.

## Supplemental information

Document S1. Figures S1–S11

Table S1. Summary of patient data and selinexor dose-response data for 44 *ex vivo* AML patient samples Tab 1: List of characteristics (e.g., age, gender, and disease characteristics) of the 44 AML patients from which the *ex vivo* samples for selinexor response were obtained. Tab 2: List of cell viability data and fitted dose-response data with determined EC50 values.

Table S2. Summary of phosphoproteomics data upon *ex vivo* selinexor treatment of AML patient samples Normalized intensities for each TMT channel are reported for all peptides with phosphorylation site localization probabilities higher than 0.75 (class 1).

Table S3. Summary of selinexor dose-response data for 15 additional *ex vivo* AML patient samples and single-shot proteome data for 30 *ex vivo* AML patient samples and AML cell lines Tab 1: Cell viability data and fitted dose-response data with determined EC50 values. Tab 2: Normalized, imputed log2-transformed LFQ intensities for 5,917 identified and quantified proteins.

Table S4. Summary of phosphoproteomics data upon selinexor treatment of AML cell lines Normalized intensities for each TMT channel are reported for all peptides with phosphorylation site localization probabilities higher than 0.75 (class 1).

Table S5. Summary of spatial proteomics data Normalized, log2-transformed, and imputed LFQ intensities for each fraction (1–6) for PL-21 and MV-4-11 after treatment with DMSO, selinexor, MK-2206, or the combination.

Table S6. Summary of spatial phosphoproteomics data Normalized, log2-transformed, and imputed LFQ intensities for each fraction (1–6) for PL-21 and MV-4-11 after treatment with DMSO, selinexor, MK-2206, or the combination. Includes peptides with a site localization probability of more than 0.75 for at least one condition.

Document S2. Article plus supplemental information
