# Supplementary material for: Phosphoproteomics of primary AML patient samples reveals rationale for AKT combination therapy and p53 context to overcome selinexor resistance
Source: Cell Rep. 2022 Aug 9;40(6):111177. doi: 10.1016/j.celrep.2022.111177 (PMC9380259; doi:10.1016/j.celrep.2022.111177)
Supplement: Document S1. Figures S1–S11 [file mmc1.pdf]

Cell Reports, Volume *40*

Supplemental information

Phosphoproteomics of primary AML patient samples reveals rationale for AKT combination therapy and p53 context to overcome selinexor resistance

Kristina B. Emdal, Nicolàs Palacio-Escat, Caroline Wigerup, Akihiro Eguchi, Helén Nilsson, Dorte B. Bekker-Jensen, Lars Rönnstrand, Julhash U. Kazi, Alexandre Puissant, Raphaël Itzykson, Julio Saez-Rodriguez, Kristina Masson, Peter Blume-Jensen, and Jesper V. Olsen

Supplementary Figures and Legends:

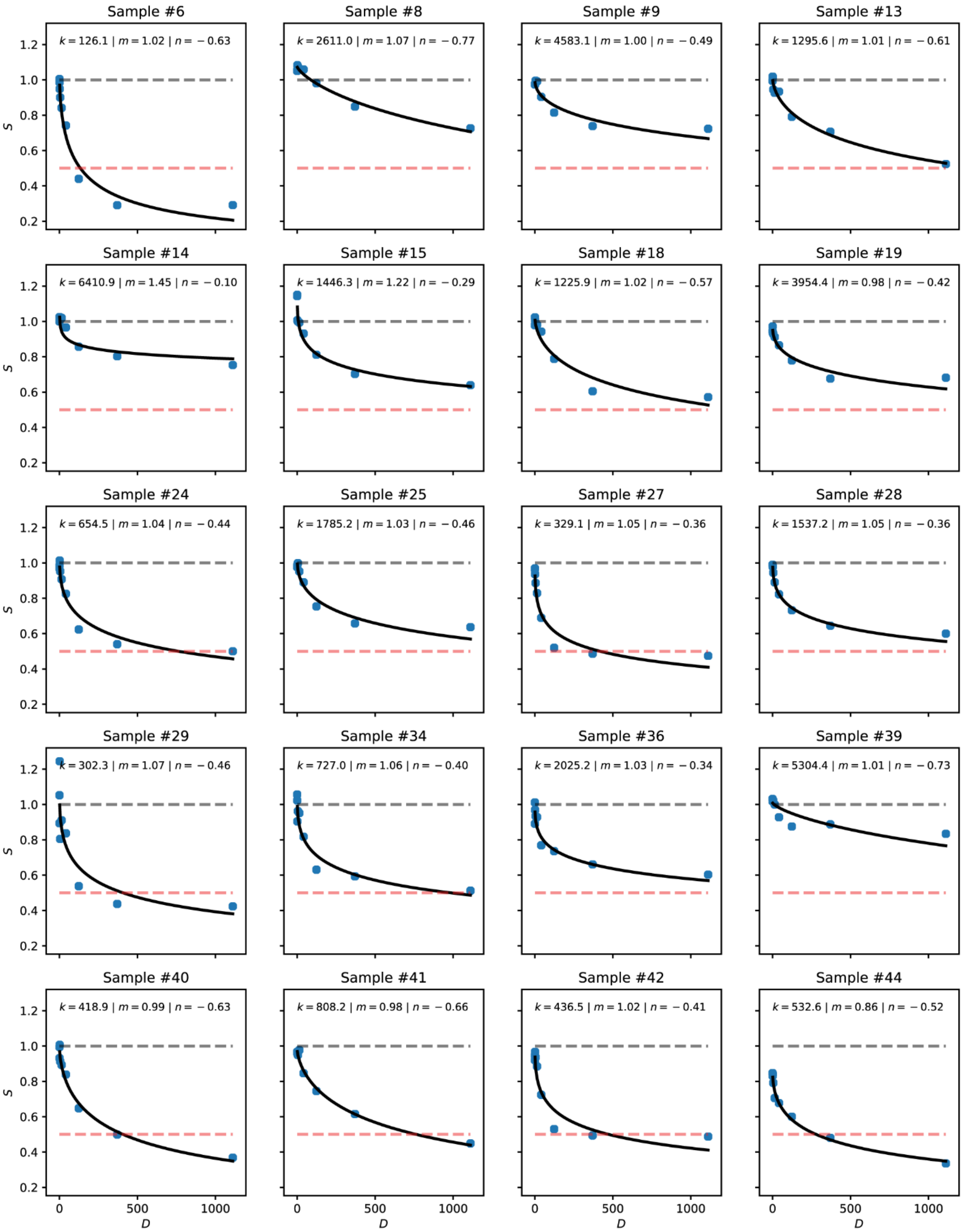

Figure S1. Selinexor dose-response cell viability curves for AML *ex vivo* patient samples included in phosphoproteomics analysis, Related to Figure 1. Cells from AML patients were treated *ex vivo* with selinexor or DMSO as control and cell viability was measured after 48 hours (n = 4 technical replicates each dose). Mean dose-response data was fitted to a dose response curve (modified Hill function) and EC50 was calculated for each sample (as shown in Fig. 1D). Dashed lines are plotted for reference, 100% cell viability in black and 50% in red.

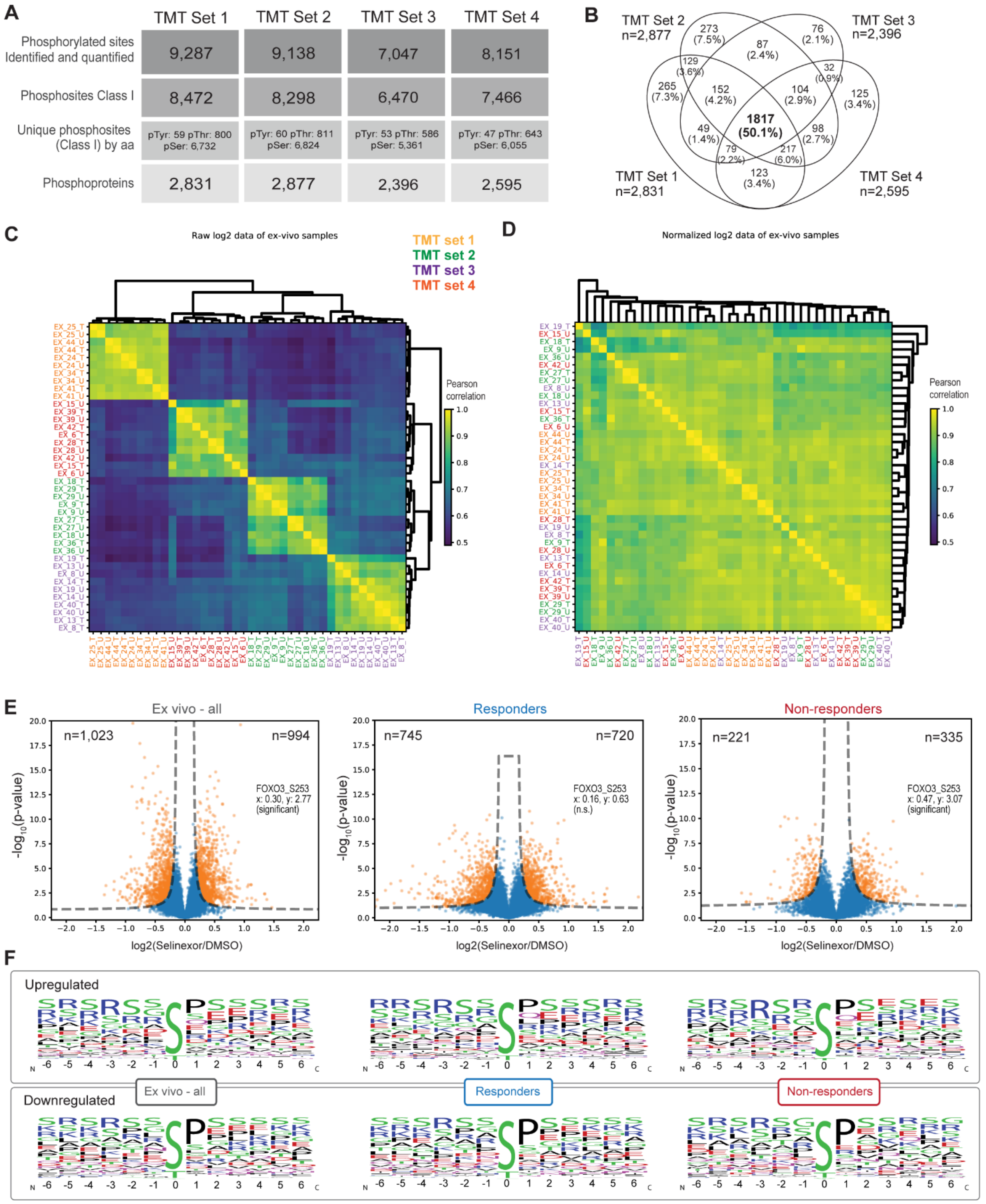

Figure S2. Phosphoproteomics of selinexor response in AML *ex vivo* patient samples, Related to Figure 1. (**A**) Summary of the number of identified phosphorylated peptides and phosphoproteins in each of the four TMT sets. (**B**) Overlap in phosphoproteins identified and quantified in the four

analyzed TMT-multiplex sets. (**C-D**) Heatmaps of the Pearson correlation coefficients comparing the four analyzed TMT-multiplex phosphoproteomics *ex vivo* data sets before (C) and after (D) normalization and batch corrections. (**E**) Volcano plots of $-\log_{10}$ transformed false discovery rate (FDR)–corrected *p*-values versus $\log_2$(fold change) of phosphorylation site intensities measured by TMT multiplexing analysis and MS. Fold change represents selinexor treatment (1 µM for 6 hours) versus DMSO control of *ex vivo* grown AML cells. Data is represented as the overall analysis (n = 20) (left panel), the group of responders (n = 9) (middle panel) and the group of non-responders (n = 11) (right panel). FOXO3 coordinates and regulation are highlighted. Two-sided t-test was performed and a site is considered significant for FDR < 0.05 and hyperbolic curve threshold of s0 = 0.1 using Perseus software. (**F**) Sequence motif enrichment analysis by WebLogo of the ±6 amino acid residues flanking the regulated phosphorylation site. Data is represented for the overall analysis (n = 20) (left panel), the group of responders (n = 9) (middle panel) and the group of non-responders (n = 11) (right panel).

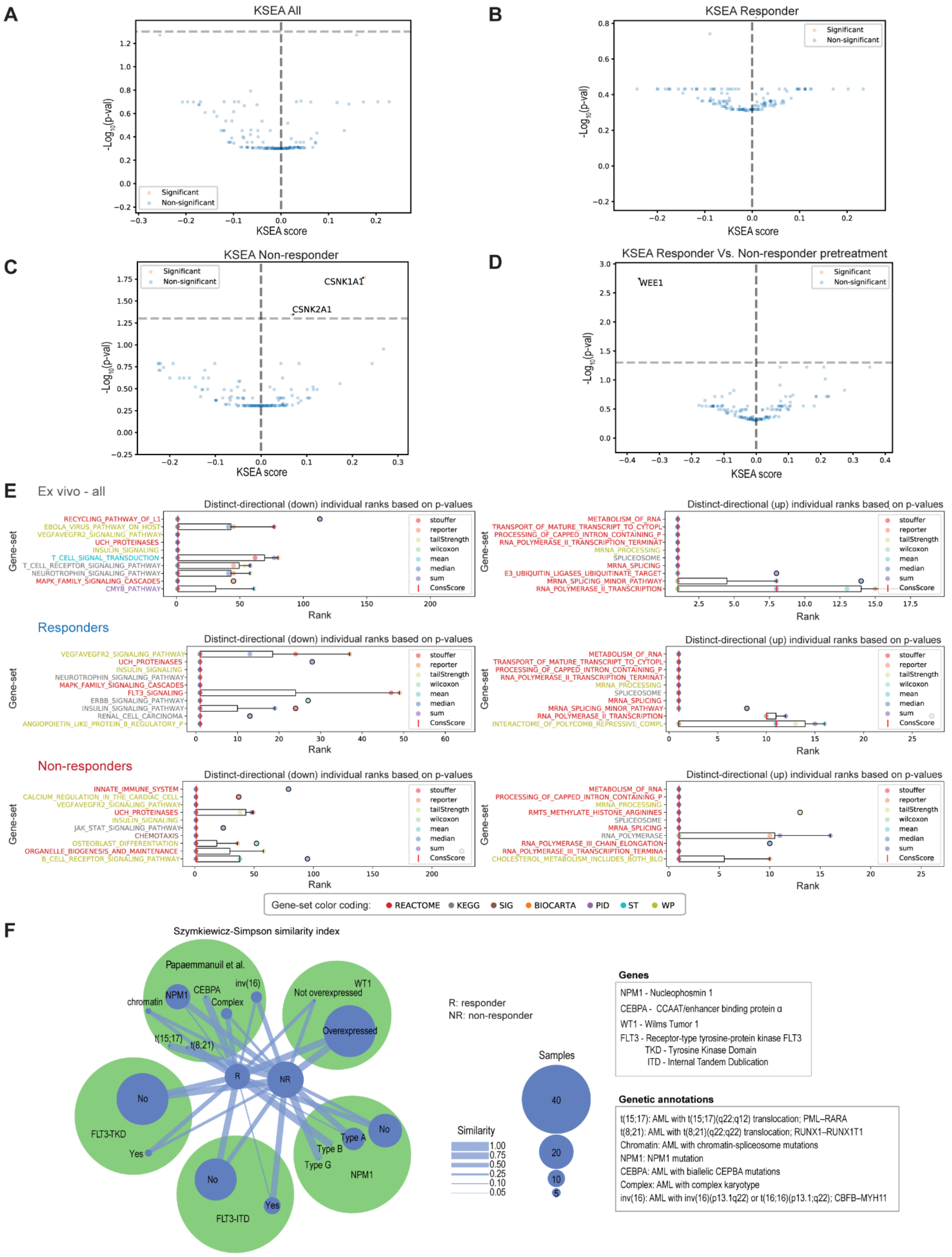

A
KSEA All
-Log10(p-val)
KSEA score
Significant
Non-significant
B
KSEA Responder
C
KSEA Non-responder
CSNK1A1
CSNK2A1
D
KSEA Responder Vs. Non-responder pretreatment
WEE1
E
Ex vivo - all
Responders
Non-responders
Distinct-directional (down) individual ranks based on p-values
Distinct-directional (up) individual ranks based on p-values
Gene-set
Rank
stouffer
reporter
tailStrength
wilcoxon
mean
median
sum
ConsScore
RECYCLING_PATHWAY_OF_L1
EBOLA_VIRUS_PATHWAY_ON_HOST
VEGFAVEGFR2_SIGNALING_PATHWAY
UCH_PROTEINASES
INSULIN_SIGNALING
T_CELL_SIGNAL_TRANSDUCTION
T_CELL_RECEPTOR_SIGNALING_PATHWAY
NEUROTROPHIN_SIGNALING_PATHWAY
MAPK_FAMILY_SIGNALING_CASCADES
CMYB_PATHWAY
METABOLISM_OF_RNA
TRANSPORT_OF_MATURE_TRANSCRIPT_TO_CYTOPL
PROCESSING_OF_CAPPED_INTRON_CONTAINING_P
RNA_POLYMERASE_II_TRANSCRIPTION_TERMINAT
MRNA_PROCESSING
SPLICEOSOME
MRNA_SPLICING
E3_UBIQUITIN_LIGASES_UBIQUITINATE_TARGET
MRNA_SPLICING_MINOR_PATHWAY
RNA_POLYMERASE_II_TRANSCRIPTION
FLT3_SIGNALING
ERBB_SIGNALING_PATHWAY
INSULIN_SIGNALING_PATHWAY
RENAL_CELL_CARCINOMA
ANGIOPOIETIN_LIKE_PROTEIN_8_REGULATORY_P
INTERACTOME_OF_POLYCOMB_REPRESSIVE_COMPL
INNATE_IMMUNE_SYSTEM
CALCIUM_REGULATION_IN_THE_CARDIAC_CELL
JAK_STAT_SIGNALING_PATHWAY
CHEMOTAXIS
OSTEOBLAST_DIFFERENTIATION
ORGANELLE_BIOGENESIS_AND_MAINTENANCE
B_CELL_RECEPTOR_SIGNALING_PATHWAY
RMTS_METHYLATE_HISTONE_ARGININES
RNA_POLYMERASE
RNA_POLYMERASE_III_CHAIN_ELONGATION
RNA_POLYMERASE_III_TRANSCRIPTION_TERMINA
CHOLESTEROL_METABOLISM_INCLUDES_BOTH_BLO
Gene-set color coding: REACTOME KEGG SIG BIOCARTA PID ST WP
F
Szymkiewicz-Simpson similarity index
Papaemmanuil et al.
chromatin
NPM1
CEBPA
Complex
inv(16)
t(15;17)
t(8;21)
WT1
Not overexpressed
Overexpressed
R
NR
No
FLT3-TKD
Yes
Type A
Type B
Type G
NPM1
FLT3-ITD
R: responder
NR: non-responder
Samples
40
20
10
5
Similarity
1.00
0.75
0.50
0.25
0.10
0.05
Genes
NPM1 - Nucleophosmin 1
CEBPA - CCAAT/enhancer binding protein α
WT1 - Wilms Tumor 1
FLT3 - Receptor-type tyrosine-protein kinase FLT3
TKD - Tyrosine Kinase Domain
ITD - Internal Tandem Dublication
Genetic annotations
t(15;17): AML with t(15;17)(q22;q12) translocation; PML–RARA
t(8;21): AML with t(8;21)(q22;q22) translocation; RUNX1–RUNX1T1
Chromatin: AML with chromatin-spliceosome mutations
NPM1: NPM1 mutation
CEBPA: AML with biallelic CEPBA mutations
Complex: AML with complex karyotype
inv(16): AML with inv(16)(p13.1q22) or t(16;16)(p13.1;q22); CBFB–MYH11

Figure S3. Kinase-substrate and gene-set enrichment analysis of AML *ex vivo* phosphoproteomics data and genetic annotation patient samples and correlation to selinexor response, Related to Figure 1. (**A-D**) Kinase-substrate enrichment analysis (KSEA) of the *ex vivo* samples for the overall analysis (A), responders (B), and non-responders (C) when comparing treated (1μM selinexor for 6h) vs. untreated (DMSO), (D) shows the KSEA results of responder samples when compared to non-responders untreated (DMSO). Dots in orange with labels are the significantly enriched kinases. A negative KSEA score denotes downregulated kinases and positive scores denote up-regulated kinases according to the regulation of their target phosphosites. Significance threshold was set to $P < 0.05$. (**E**) Gene-set enrichment analysis (GSEA) scores for the top 10 ranked gene sets among the down- (left panels) and up-regulated (right panels) in the overall analysis (top), responders (middle) and non-responders (bottom). The gene sets are ranked according to the consensus rank (median) across different statistical methods used for the enrichment (colored dots). (**F**) Szymkiewicz-Simpson index (overlap coefficient) of genetic annotations and response groups. Also known as overlapping coefficient, the Szymkiewicz-Simpson index is defined as the ratio between the size of the intersection between two sets divided by the size of the smaller one of the sets. Genetic annotations considered are known alterations in AML and described in the figure (Papaemmanuil et al., 2016). Circle sizes represent the number of samples in each group and line thickness the overlap coefficient value.

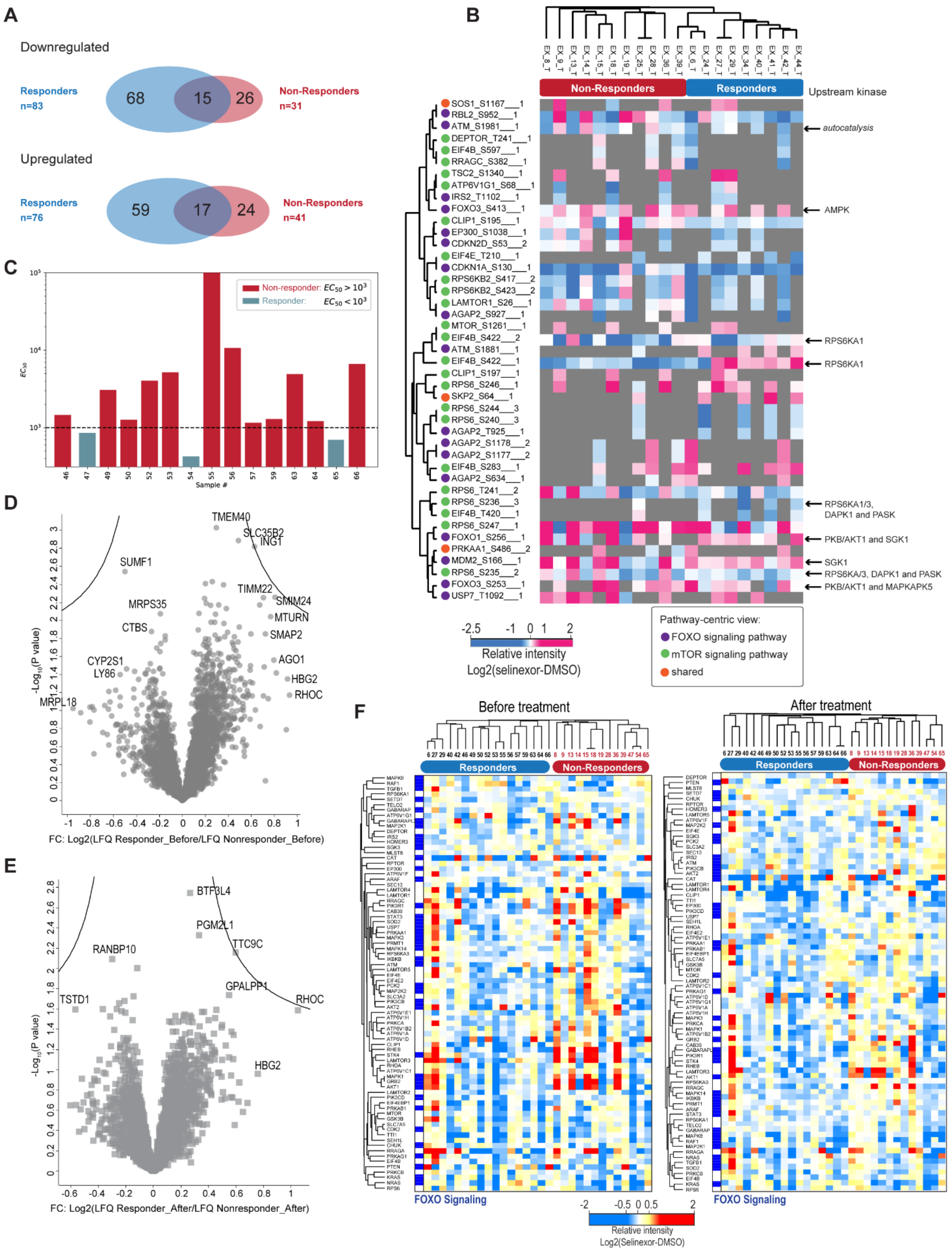
A
Downregulated
Responders n=83
68
15
26
Non-Responders n=31
Upregulated
Responders n=76
59
17
24
Non-Responders n=41
B
Non-Responders
Responders
Upstream kinase
autocatalysis
AMPK
RPS6KA1
RPS6KA1
RPS6KA1/3, DAPK1 and PASK
PKB/AKT1 and SGK1
SGK1
RPS6KA/3, DAPK1 and PASK
PKB/AKT1 and MAPKAPK5
Relative intensity
Log2(selinexor-DMSO)
Pathway-centric view:
FOXO signaling pathway
mTOR signaling pathway
shared
C
Non-responder: $EC_{50} > 10^3$
Responder: $EC_{50} < 10^3$
EC_{50}
Sample #
D
-Log10(P value)
FC: Log2(LFQ Responder_Before/LFQ Nonresponder_Before)
E
-Log10(P value)
FC: Log2(LFQ Responder_After/LFQ Nonresponder_After)
F
Before treatment
After treatment
Responders
Non-Responders
FOXO Signaling
Relative intensity
Log2(Selinexor-DMSO)

Figure S4. Pathway-centric focus of the phosphoproteome and proteome of *ex vivo* AML patient samples, Related to Figure 2. (**A**) Overlap in number of functional phosphorylation sites (score >0.6) among down- (upper panel) and upregulated (lower panel) between responders and non-responders. (**B**) Heatmap of the log2(FC) for all regulated phosphorylation sites identified for responders and non-responders assigned to the KEGG pathways: FOXO signaling (KEGG database identifier: 04068) and mTOR signaling (KEGG database identifier: 04150). Grey color indicates missing quantification by mass spectrometry and suffixes _1, _2, _3 refer to the whether the quantification was derived from mono-, doubly- or triply-phosphorylated peptides. (**C**) Selinexor EC50 values for 15 *ex vivo* AML patient samples. Values were extracted from dose-response models based on cell viability after treatment (48h) with selinexor (See also Fig. S5 for dose-response curves of the 15 patient samples). A binary cut-off of 1000 nM (dashed line) was used to determine selinexor sensitivity. Patients with EC50 < 1000 nM were considered selinexor responders (n = 3) and patients with EC50 > 1000 nM were considered non-responders (n = 12). (**D-E**) Volcano plots of $-\log_{10}$ transformed false discovery rate (FDR)–corrected *P*-values versus $\log_2$(fold change) of protein LFQ intensities from DIA-based single-shot proteome MS analysis of 30 *ex vivo* AML patient samples. Fold change represents pre-treatment (C) (before) and post-treatment (D) (after; 1 µM selinexor for 6 hours) of responders versus non-responders. Two-sided t-test was performed and a protein is considered significant for FDR < 0.05 and hyperbolic curve threshold of s0 = 0.1 using Perseus software. (**F**) Heatmap of the log2-transformed pre- (before) and post-treatment (after) LFQ intensities for proteins identified for responders and non-responders and assigned to the KEGG pathways: FOXO signaling (KEGG database identifier: 04068; in blue) and mTOR signaling (KEGG database identifier: 04150).

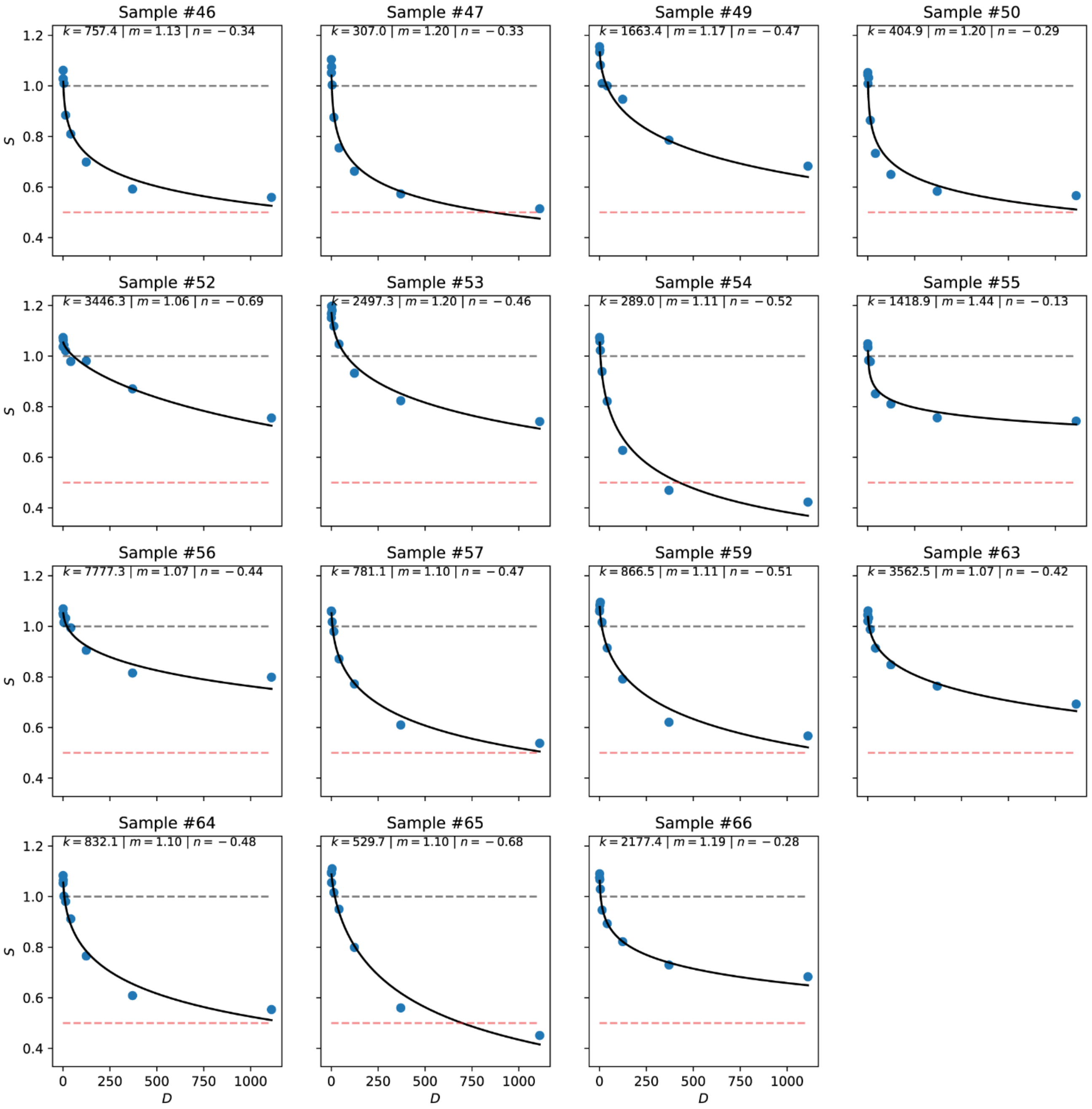

Figure S5. Selinexor dose-response cell viability curves for 15 additional AML *ex vivo* patient samples, Related to 2. Cells from AML patients were treated *ex vivo* with selinexor or DMSO as control and cell viability was measured after 48 hours (n = 4 technical replicates each dose). Mean dose-response data was fitted to a dose response curve (modified Hill function) and EC50 was calculated for each sample (as shown in Fig. S4C and Table S3). Dashed lines are plotted for reference, 100% cell viability in black and 50% in red.

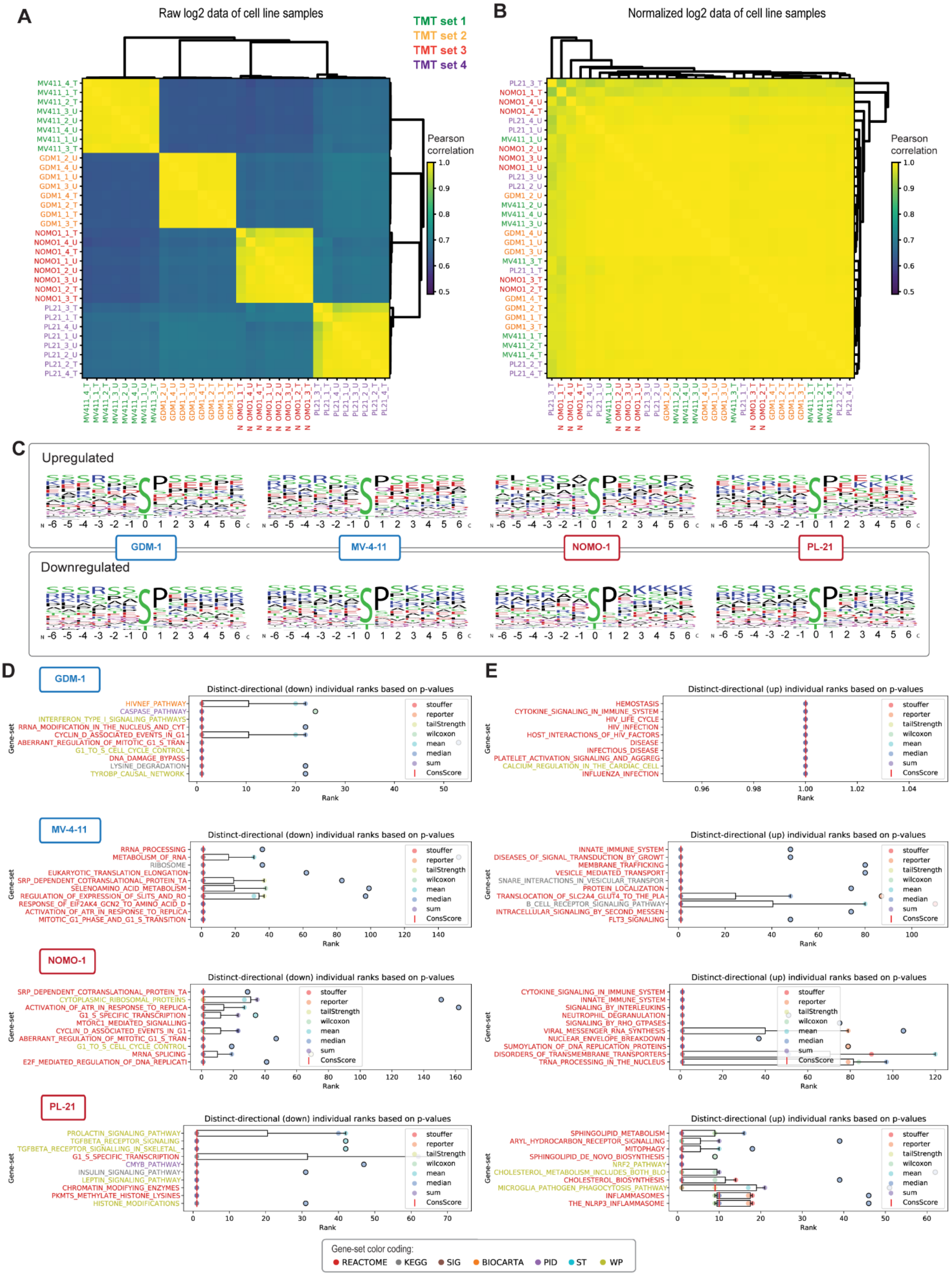
A
Raw log2 data of cell line samples
TMT set 1
TMT set 2
TMT set 3
TMT set 4
Pearson correlation
B
Normalized log2 data of cell line samples
Pearson correlation
C
Upregulated
Downregulated
GDM-1
MV-4-11
NOMO-1
PL-21
D
E
Distinct-directional (down) individual ranks based on p-values
Distinct-directional (up) individual ranks based on p-values
Gene-set
Rank
stouffer
reporter
tailStrength
wilcoxon
mean
median
sum
ConsScore
Gene-set color coding:
REACTOME
KEGG
SIG
BIOCARTA
PID
ST
WP

Figure S6. Effects of normalization of AML cell line phosphoproteome data, sequence motif and gene-set enrichment analysis, Related to Figure 3. (**A-B**) Heatmaps of the Pearson correlation coefficients comparing the four analyzed TMT-multiplex phopshoproteomics data sets obtained from AML cell lines before (A) and after (B) normalization and batch correction. (**C**) Sequence motif enrichment analysis by WebLogo of the ±6 amino acid residues flanking the regulated phosphorylation site. Data is represented for the group of up- and downregulated phosphorylation sites for each of the AML cell lines as indicated. (**D-E**) Gene-set enrichment analysis (GSEA) scores for the top 10 ranked gene sets among the down- (D) and upregulated (E) for each AML cell line as indicated. The gene sets are ranked according to the consensus rank (median) across different statistical methods used for the enrichment (colored dots). See also Table S4.

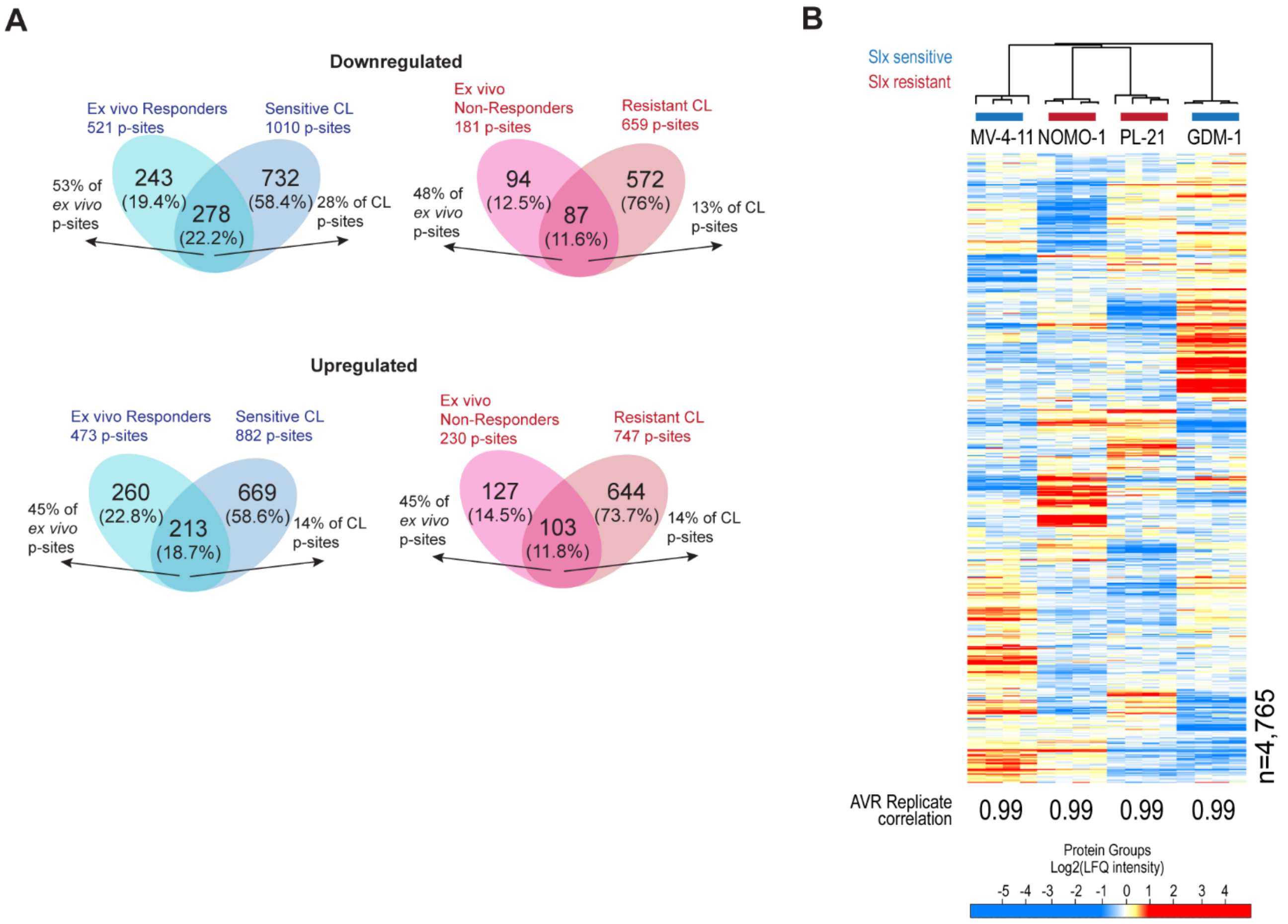

Figure S7. Phosphoregulation overlap with *ex vivo* phosphoproteome and proteome profiling of AML cell lines, Related to Figure 4. (**A**) Overlap in number of identified phosphorylation sites (p-sites) regulated (downregulated: left, upregulated: right) among *ex vivo* responders and sensitive AML cell lines (blue) and non-responders and resistant AML cell lines (red). (**B**) Hierarchical clustering of log2-transformed LFQ intensities of proteins (n=4,765; quantified with >2 peptides) identified in the single-shot proteome analysis of MV-4-11, NOMO-1, PL-21, and GDM-1 cells (n = 4 bioreps for each cell line). See also Table S3.

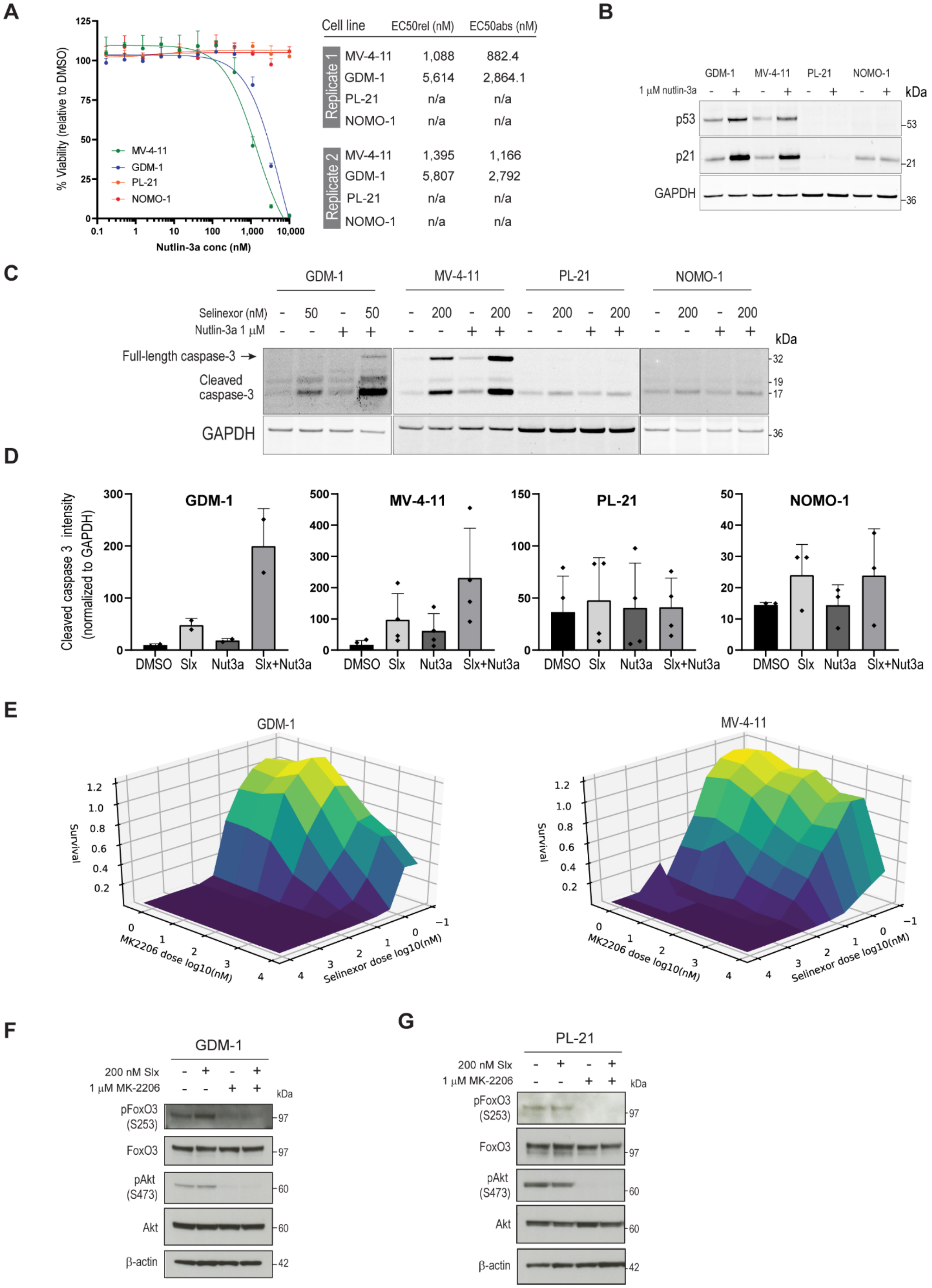

	Cell line	EC50rel (nM)	EC50abs (nM)
Replicate 1	MV-4-11	1,088	882.4
	GDM-1	5,614	2,864.1
	PL-21	n/a	n/a
	NOMO-1	n/a	n/a
Replicate 2	MV-4-11	1,395	1,166
	GDM-1	5,807	2,792
	PL-21	n/a	n/a
	NOMO-1	n/a	n/a

Figure S8. Combination treatment of selinexor sensitive and resistant AML cell lines, Related to Figure 5. (**A**) Dose-response cell viability assay for MV-4-11, GDM-1, PL-21, and NOMO-1. Cells were treated for 72 hours with 11 different concentrations of nutlin-3a (n = 4 technical replicates each dose). DMSO was used as control and estimated relative and absolute EC50 values each of the cell lines was calculated (n = 2 bioreps). (**B**) Western blot of lysates of MV-4-11, GDM-1, PL-21, and NOMO-1 treated with DMSO or nutlin-3a (1 μM) for 24 hours and immunoblotted for p53, p21 and β-actin (for reference) (n = 2). (**C-D**) Western blot (C) lysates from MV-4-11, GDM-1, PL-21, and NOMO-1 treated with selinexor (GDM-1: 50 nM, rest 200 nM) or nutlin-3a (1 μM) alone or the combination for 24 hours and immunoblotted for cleaved caspase-3 and GAPDH (for reference). Blots are quantified in (D) and bar graphs represent mean +/- S.D. (n = 3, except GMD-1: n = 2). **(E)** GDM-1 and MV-4-11 cells were treated with selinexor and MK-2206 alone or in combination and cell viability was measured after 72 hours. 3D plots show the mean relative survival (color scale, z-axis) against selinexor and MK-2206 doses (x and y axes). (**F-G**) Western blot of lysates from GDM-1 and PL-21 cells treated with selinexor (200 nM), MK-2206 (1 μM) alone or the combination for 24 hours and immunoblotted for indicated antibodies and β-actin (for reference). Blots are representative of n = 1 for GDM-1 and n = 2 for PL-21 cells.

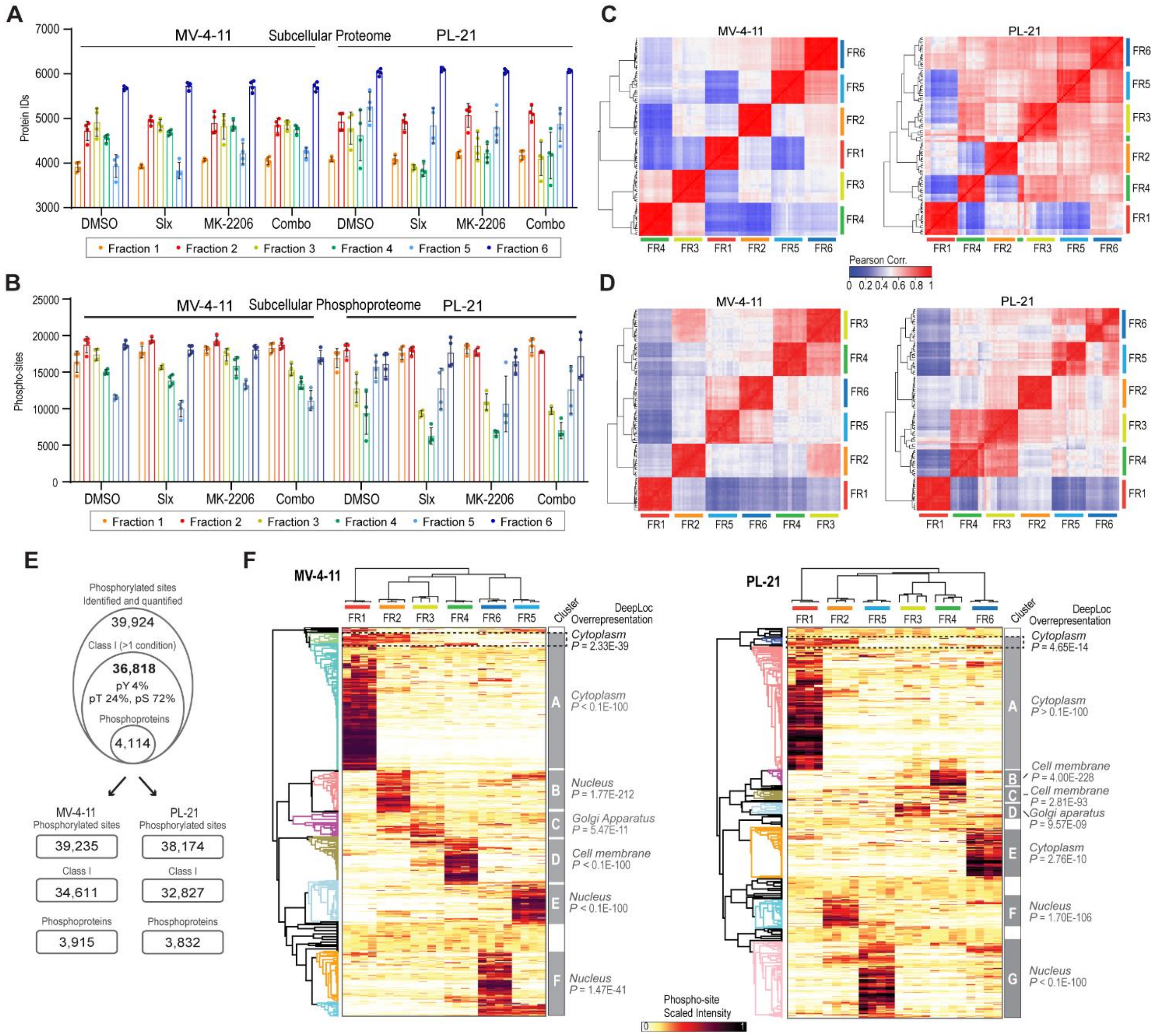

Figure S9. Subcellular (phospho)proteome analysis upon selinexor and MK-2206 single and combination treatment, Related to Figure 6. (**A-B**) Bar-plot summary of the identified proteins (A) and phosphorylation sites (B) in MV-4-11 and PL-21 cells as average of n = 4 biological replicates per fraction. Data represent mean ± SD across fractions 1-6 for each treatment condition. (**C-D**) Heatmaps of Pearson's correlation coefficient (R-value) from MV-4-11 (left) and PL-21 (right) cells for the subcellular proteome (C) and phosphoproteome (D) analysis of in total 4 treatment conditions; DMSO, selinexor, MK-2206 and combination (in n = 4 replicates) across 6 fractions. (**E**) Overview of the spatial phosphoproteome data summarizing the number of identified phosphorylation sites and phosphoproteins derived from the analysis of MV-4-11 and PL-21 cells. (**F**) Heatmap of scaled intensities per fraction of the subcellular phosphoproteome of MV-4-11 (left) and PL-21 (right) cells

(DMSO-treated cells; n = 4 independent experiments), showing phosphorylation site and sample clustering. See also Table S5 and Table S6.

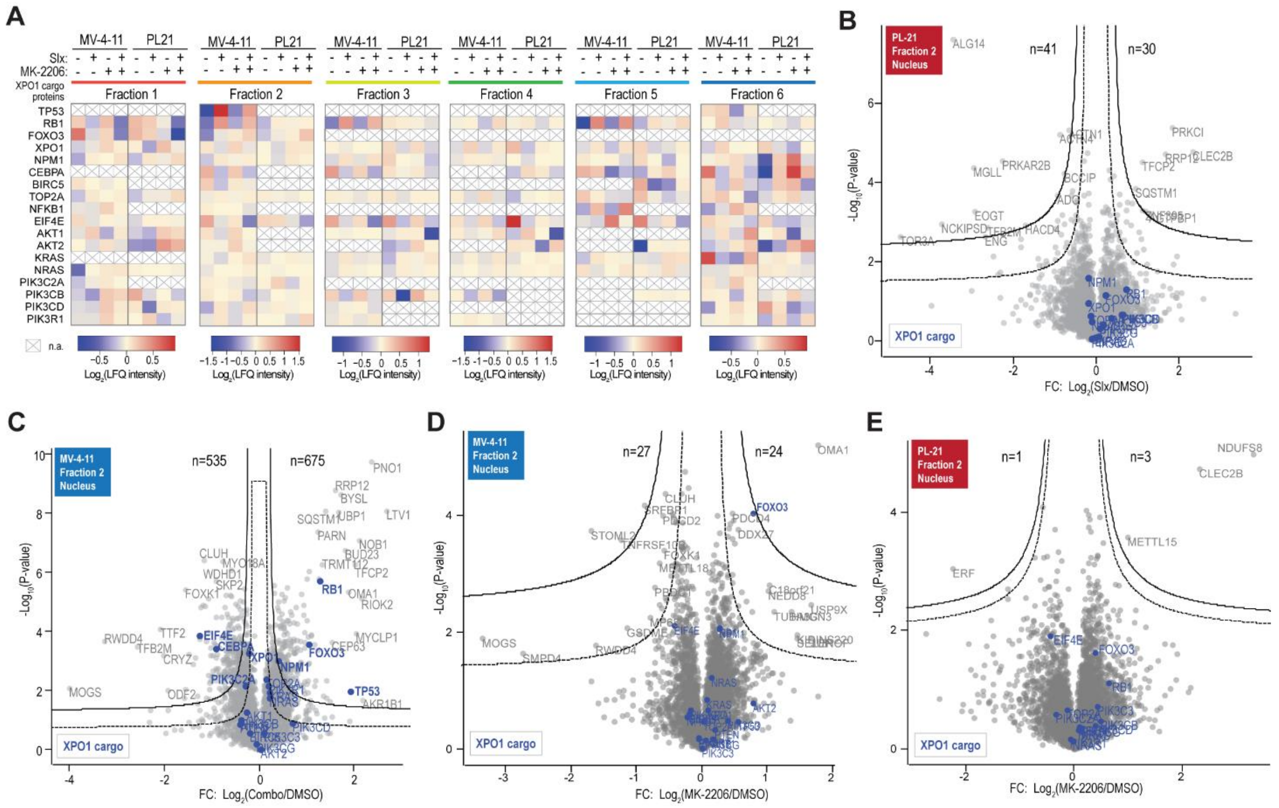

Figure S10. Subcellular proteome response to selinexor and MK-2206 single and combination treatment in MV-4-11 and PL-21 cells, Related to Figure 6. (**A**) Heatmap representation of a selection of XPO1 cargo with average log2-transformed LFQ intensities of n = 4 replicates per treatment condition. (**B-E**) Volcano plots of $-\log_{10}$ transformed false discovery rate (FDR)–corrected *P*-values versus $\log_2$(fold change) of LFQ protein intensities measured by MS in fraction 2. Fold change represents selinexor treatment (1 µM for 6 hours) versus DMSO control in PL-21 (B), the combination (selinexor and MK-2206) versus DMSO in MV-4-11 cells (C) and MK-2206 treatment alone (1 µM for 6 hours) versus DMSO control in MV-4-11 (D) and PL-21 (E) cells. A two-sided t-test was performed and a protein is considered significant for FDR < 0.05 (dotted line) and FDR < 0.01 (solid line) and hyperbolic curve threshold of s0 = 0.1 using Perseus software. A selection of known XPO1 cargo is highlighted in blue. See also Table S5.

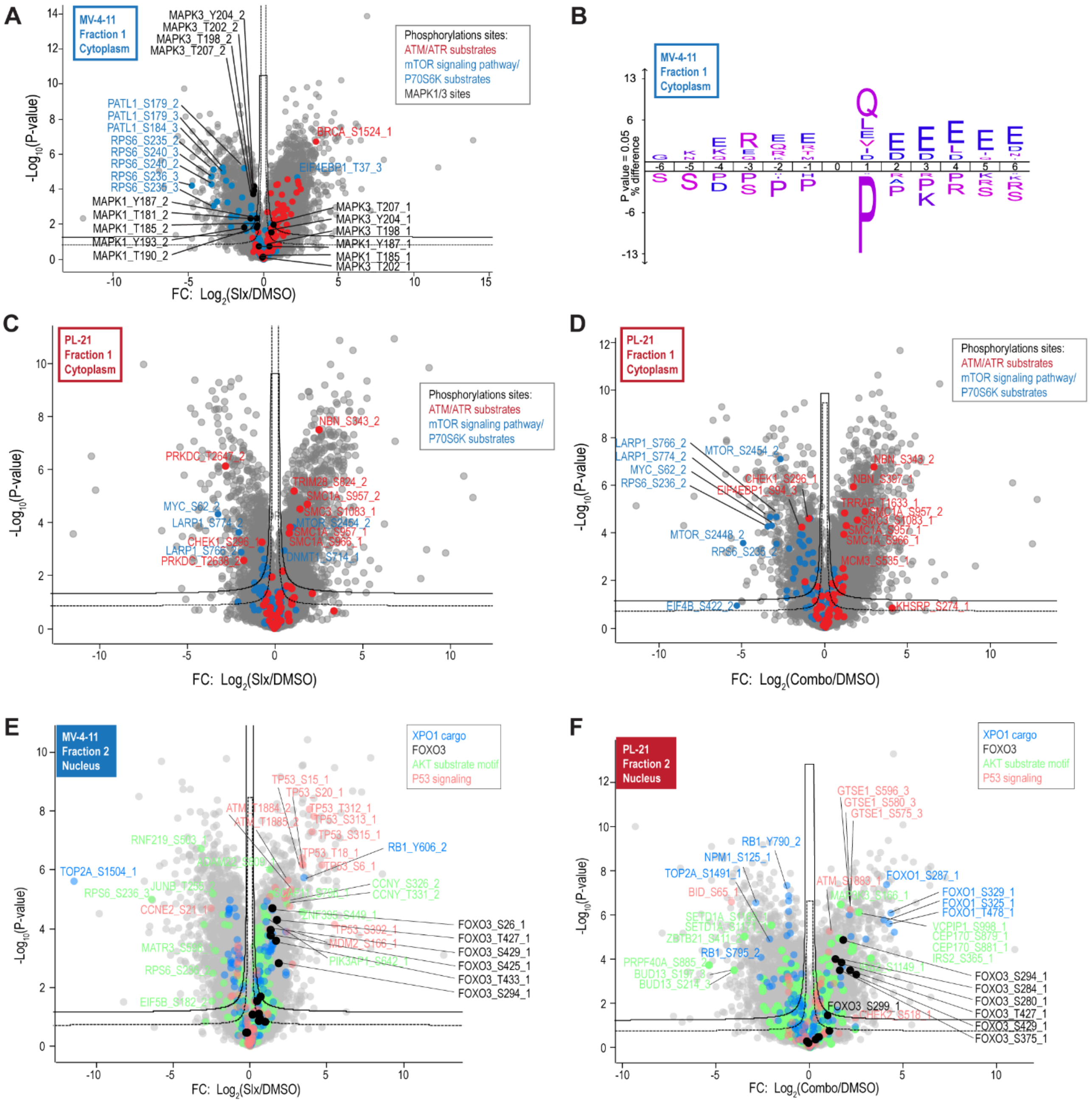

Figure S11. Subcellular phosphoproteome response to selinexor in MV-4-11 cells is recapitulated by drug synergy combination with MK-2206 in PL-21 cells, Related to Figure 6. (**A, C-F**) Volcano plots showing differentially regulated phosphorylation sites. Significance was deemed by a two-sided t-test (FDR < 0.05 (dotted line), FDR < 0.01 (solid line), s0 = 0.1) using Perseus software. Fold change represents selinexor treatment versus DMSO control in MV-4-11 cells in fraction 1 (A). (**B**) Sequence motif analysis of the upregulated phosphorylation sites compared to sites that are downregulated in (A). (**C-F**) Volcano plots of fraction 1 in PL-21 cells with fold change represented by selinexor treatment (C) and synergy combination (D) versus DMSO control. Volcano

plots of fraction 2 (E-F) show fold change for selinexor treatment versus DMSO in MV-4-11 cells (E) and synergy combination versus DMSO in PL-21 cells (F). See also Table S6.
